# A circumpolar dust conveyor in the glacial Southern Ocean

**DOI:** 10.1038/s41467-020-18858-y

**Published:** 2020-11-09

**Authors:** Torben Struve, Katharina Pahnke, Frank Lamy, Marc Wengler, Philipp Böning, Gisela Winckler

**Affiliations:** 1 Marine Isotope Geochemistry, Institute for Chemistry and Biology of the Marine Environment (ICBM), University of Oldenburg, 26129 Oldenburg, Germany; 2 Alfred Wegener Institute for Polar and Marine Research, 27568 Bremerhaven, Germany; 3 Lamont-Doherty Earth Observatory of Columbia University, Palisades, New York 10964 USA; 4 Department of Earth and Environmental Sciences, Columbia University, New York, New York 10027 USA

**Keywords:** Palaeoceanography, Palaeoclimate, Geochemistry

## Abstract

The increased flux of soluble iron (Fe) to the Fe-deficient Southern Ocean by atmospheric dust is considered to have stimulated the net primary production and carbon export, thus promoting atmospheric CO_2_ drawdown during glacial periods. Yet, little is known about the sources and transport pathways of Southern Hemisphere dust during the Last Glacial Maximum (LGM). Here we show that Central South America (~24‒32°S) contributed up to ~80% of the dust deposition in the South Pacific Subantarctic Zone via efficient circum-Antarctic dust transport during the LGM, whereas the Antarctic Zone was dominated by dust from Australia. This pattern is in contrast to the modern/Holocene pattern, when South Pacific dust fluxes are thought to be primarily supported by Australian sources. Our findings reveal that in the glacial Southern Ocean, Fe fertilization critically relies on the dynamic interaction of changes in dust-Fe sources in Central South America with the circumpolar westerly wind system.

## Abstract (web summary)

Dust deposition brings iron that fuels ocean productivity, a connection impacting climate over geological time. Here the authors use sediment cores to show that in contrast to dynamics today, during the last glacial maximum westerly winds shuttled dust from Australia and South America around Antarctica and into the South Pacific.

## Introduction

Air trapped in Antarctic ice indicates that glacial atmospheric CO_2_ concentrations were lower by about 80‒100 p.p.m. compared to interglacial levels over the past 800,000 years^1^. Past variability in the atmospheric CO_2_ concentrations was synchronous with variations in air temperature and dust deposition over Antarctica on glacial–interglacial to millennial time scales^1–3^.

It has been hypothesized that physical and biogeochemical feedbacks in the Southern Hemisphere enhanced the net primary productivity and carbon export during glacials playing a crucial role for the observed coupling between dust, CO_2_, and temperature changes^4–7^. The deficiency of the micronutrient iron (Fe) is considered a limiting factor for phytoplankton growth in the Southern Ocean^8–10^. Martin^8^ proposed that the increased input of Fe-bearing mineral dust to the Southern Ocean would lead to increased primary productivity and enhanced oceanic sequestration of CO_2_ during past glacial periods. This relationship has been studied extensively in the modern Southern Ocean^9,10^ where the export of organic carbon from the surface to the deep ocean can be particularly efficient^11^. However, the effects on net carbon export remain equivocal, partly due to the temporal and spatial limitations of artificial Fe fertilization experiments^10,12^. More continuous natural Fe fertilization in the Southern Ocean suggests higher carbon export, but spatial limitations remain^10^.

Evidence for large-scale natural Fe fertilization experiments is preserved in the marine geological record and confirms the mechanistic link between Southern Ocean dust deposition, primary productivity, CO_2_, and temperature during the late Pleistocene glacial–interglacial cycles^5–7^. Specifically, the dust-Fe-induced increases in primary productivity enhanced nutrient consumption and export productivity (i.e., export of organic carbon) in the Southern Ocean Subantarctic Zone (SAZ) during past glacials^5,6,13^. This effect was suggested to account for a net drawdown of atmospheric CO_2_ of up to ~40 p.p.m. representing almost half of the total glacial–interglacial change^6,7,14^.

The data-based estimates of dust-Fe-induced drawdown of atmospheric CO_2_ critically rely on reconstructions of particle fluxes and nutrient consumption from the South Atlantic and extrapolation of the results to the entire Southern Ocean^5,6^. These estimates do not take into account contributions from different dust sources in the Southern Hemisphere^15,16^. However, the solubility and bioavailability of dust-borne Fe in the surface ocean is controlled by the complex interaction of multiple factors, including source area particle mineralogy^17–20^, atmospheric transport (organic complexation, (photo)chemical reactions and pH, and particle sorting)^18,21,22^, sea-ice processing^23,24^, surface ocean photochemistry, and seawater biogeochemistry (Fe chemistry, biotic processing, abundance and type of organic ligands, and particle interactions)^21,22,25^. Changes in dust provenance are primarily linked with variations in source area mineralogy and the characteristics of atmospheric particle transport influencing particle–liquid interaction in the atmosphere and in the surface ocean^18,22^.

In the present day, the largest contributions to the total dust flux in the individual sectors of the Southern Ocean are immediately upwind in Australia, South America, and South Africa following the circumpolar flow of the Southern Hemisphere Westerly Winds (SWW)^15,26,27^ (Supplementary Fig. 1). Air parcel trajectories suggest that dust emissions from all continental dust sources in the mid-latitude Southern Hemisphere, including New Zealand, can contribute to the total dust flux to the Southern Ocean^16^. However, Australia and South America are typically considered the most prominent dust sources in the Southern Hemisphere, hosting multiple dust source regions in their (semi)arid continental interior and on the eastern side of the Andes, respectively^26–29^. The modern dust emissions from terrestrial source regions in the Southern Hemisphere have been traced downwind using their distinct geochemical fingerprints^28,30–35^.

Reconstructions of dust provenance in past environments benefit from the geochemical differences between individual potential dust source areas (PSAs) and revealed that southern South American sources, and in particular Patagonia (south of 38°S), dominated the dust supply to East Antarctica during glacials^28,31,36–39^. Yet, currently available Antarctic ice-core data do not unambiguously resolve specific terrestrial sources due to geochemical similarities between important dust sources in Australia, New Zealand, and South America^31,33,37,40^, and/or analytical limitations resulting from the low abundance of lithogenic dust particles in Antarctic ice^35,38^. Previous work from the South Pacific SAZ showed that the general pattern of glacial–interglacial variability in dust deposition resembles the characteristics of Antarctic ice-core records, with higher input during glacials compared to interglacial periods^41^. The increased glacial dust supply to the South Pacific SAZ was ascribed to Australian and/or New Zealand sources synchronized with Patagonian dust emissions by large-scale common climate forcing^20,41,42^. However, relatively little is known about sources and transport of dust to the glacial South Pacific SAZ^42^ comprising the largest area for oceanic CO_2_ sequestration through Fe fertilization in the Southern Hemisphere. Hence, characterizing the sources of dust input to the South Pacific SAZ during the Last Glacial Maximum (LGM) is paramount to further understanding the role of the Southern Hemisphere dust cycle in the glacial drawdown of atmospheric CO_2_. Here we use a set of complementary, but independent geochemical tracers including rare earth elements (REEs), strontium (Sr), neodymium (Nd), and lead (Pb) isotopes to constrain sources and transport paths of dust delivered to the mid-latitude South Pacific during the LGM. Our data reveal remarkable changes in the geochemistry and spatial distribution of dust between the LGM and the Holocene, refining the existing picture of the glacial Southern Hemisphere dust cycle.

## Results and discussion

### Isotope signatures of South Pacific fine fraction sediments

**Fig. 1:**
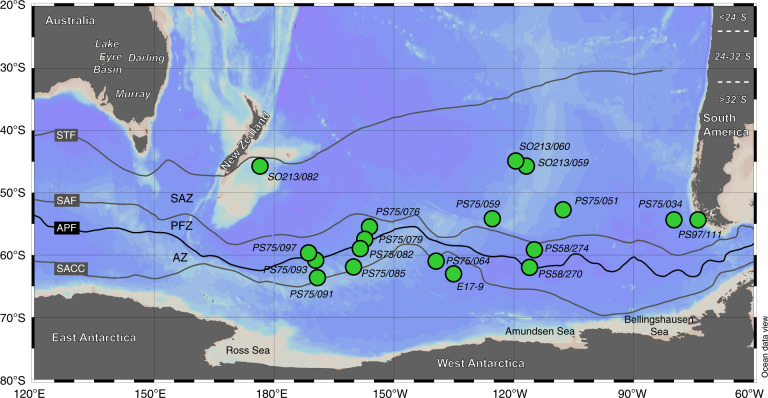
Sample locations in the South Pacific. Main terrestrial potential source areas (PSAs) indicated with white labels. South American dust sources are grouped into latitudinal bands. In the text, PSAs between 24 and 32°S are also referred to as Central South America, whereas Southern South America describes the PSAs south of 32°S. Southern Ocean fronts from ref. ^94^. STF: Subtropical Front. SAF: Subantarctic Front. APF: Antarctic Polar Front. SACC: Southern ACC Front. SAZ: Subantarctic Zone. PFZ: Polar Frontal Zone. AZ: Antarctic Zone. Map created with Ocean Data View^95^.

For this study, we selected 18 locations between ~2 and 5 km water depth covering the mid-latitude South Pacific from ~174°E to ~75°W and from ~45°S to ~63°S across the Antarctic Circumpolar Current (ACC) (Fig. 1). For each location, we typically processed five samples of the < 5 µm sediment fraction across the LGM interval between 18,000‒24,000 years before present (i.e., 18‒24 ka BP) (*n* = 85), which was identified using available age constraints (see Supplementary Data File 1).

We have chosen the < 5 µm size fraction for our dust provenance study, because the long atmospheric lifetime of this size fraction^43^ enables inter-continental (long-range) airborne transport^44^ and thus the dominant role of < 5 µm particles in the lithogenic sediment fraction of the South Pacific SAZ^45^. Furthermore, moderate changes in grain-size composition during atmospheric transport^43^ reduce possible bias from grain-size effects in the < 5 µm fraction, thus allowing direct comparison to many geochemical data sets from terrestrial PSAs. An additional asset is that the high cohesiveness of this size fraction minimizes resuspension and post-depositional transport by bottom currents^46^. The details of sample preparation and analyses are outlined in the “Methods” section. The results are reported as averages per sampling location with 2 SD across the LGM time slice (*n* = 2 ‒ 7).

**Fig. 2:**
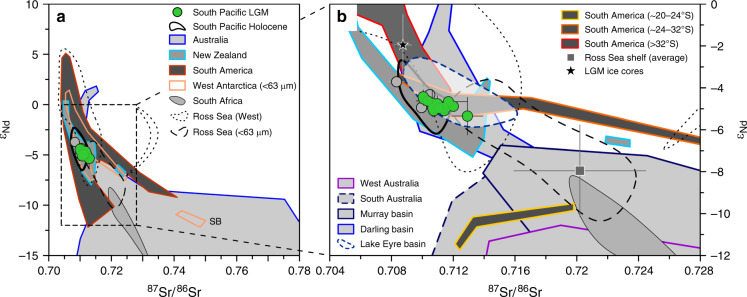
South Pacific Last Glacial Maximum fine fraction samples in Nd-Sr isotope space. Remote open ocean samples in green and locations at/near the continental margins in gray. Last Glacial Maximum (LGM) data points and error bars represent averages and their 2 SD across the LGM interval (~18‒24 ka BP). See Supplementary Fig. 1 and Supplementary Tables 2 and 3 for details and references to terrestrial source area data. **a** Overview of potential source area compositions in the Southern Hemisphere. West Antarctic and Ross Sea shelf data obtained from < 63 µm fractions^50,96^. SB: Sulzberger Bay. **b** Detailed view with South American and Australian source areas subdivided into individual provinces. Average values for Antarctic ice cores calculated from LGM age samples at Dome B and C (*ε*_Nd_ = −2.0 ± 1.5, ^87^Sr/^86^Sr = 0.7087 ± 0.0006, 2 SD, *n* = 9)^31,44^. Ross Sea shelf average^96^ is indicative of the composition of ice-rafted detritus exported into the South Pacific^47^. Note the isotopic difference of Southwest Pacific location PS75/091-3 relative to the remaining open ocean data (see Supplementary Note 5 and Supplementary Fig. 6 for further details).

**Fig. 3:**
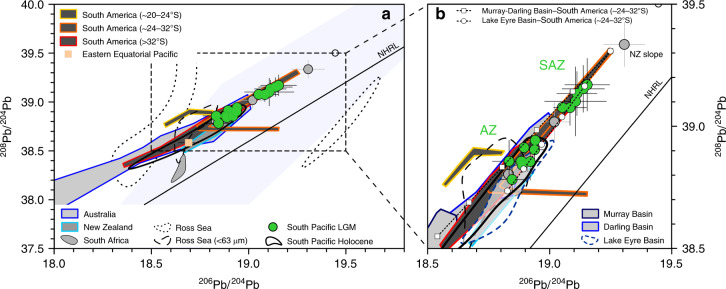
South Pacific Last Glacial Maximum fine fraction samples in ^208^Pb/^204^Pb-^206^Pb/^204^Pb space. Remote open ocean samples in green and locations at/near the continental margins in gray. Last Glacial Maximum (LGM) data points and error bars represent averages and their 2 SD across the LGM interval (~18–24 ka BP). Black ring indicates coretop sample PS75/105-1 from the New Zealand shelf^34^. See Supplementary Fig. 1 and Supplementary Tables 2 and 3 for details and references to potential source area (PSA) data. **a** Overview of Southern Hemisphere PSA compositions. Average LGM value for the Eastern Equatorial Pacific from ref. ^71^. Light blue shading indicates Dome C ice-core data^38^. NHRL: Northern Hemisphere Reference Line^97^. **b** Detailed view including endmember mixing models. Filled white symbols represent 20% increments of mixing of Central South America (24‒32°S) with Lake Eyre (circles) and Murray–Darling endmembers (squares), respectively. Australia is subdivided into individual provinces. AZ: Antarctic Zone. SAZ: Subantarctic Zone. It is noteworthy that one Central South American sample is depleted in ^208^Pb relative to ^207^Pb (see Supplementary Fig. 4 for comparison), and that Lake Eyre Basin data were obtained from bulk sample material^38^.

We obtained Nd isotope compositions (expressed as *ε*_Nd_; see “Methods” for more details) between *ε*_Nd_ = −3.7 ± 0.5 and −5.3 ± 0.9, and Sr isotope compositions ranging from ^87^Sr/^86^Sr = 0.7083 ± 0.0005 to 0.7129 ± 0.0010 (Fig. 2 and Supplementary Data File 2). The Pb isotope results for ^206^Pb/^204^Pb range from 18.831 ± 0.027 to 19.306 ± 0.085 with a tendency towards more radiogenic Pb isotope compositions and higher variability at locations in the South Pacific SAZ compared to the Antarctic Zone (AZ) (Fig. 3 and Supplementary Data File 2). Here we focus on the samples from the remote open ocean (*n* = 70). For more details on locations near the continental margins and PS75/034 close to South America, see Supplementary Note 3. All PSA data included in the discussion below were obtained from size fractions typical for far-traveled mineral dust (< 2, < 5, and < 10 µm) if not indicated otherwise (see Supplementary Fig. 1 and Supplementary Table 3 for more details).

### Mode of fine particle transport to the glacial South Pacific

A prominent characteristic of our LGM < 5 µm fraction data set is a relatively narrow range of Nd and Sr isotope compositions (*ɛ*_Nd_ of −4.9 ± 0.6 and ^87^Sr/^86^Sr = 0.7111 ± 0.0015, *n* = 70, 2 SD of the remote open ocean sample population). This is in contrast to the large range of Nd-Sr isotope compositions of Southern Hemisphere PSAs in Australia, New Zealand, South America, South Africa, and Antarctica (Fig. 2). Of these PSAs, Antarctica is the only source region which releases significant amounts of lithogenic material that can reach the mid-latitude South Pacific by multiple modes of transport including rafting ice, (re)suspension by bottom currents, turbidity flows, and dust^24,34,42,47,48^. Identifying lithogenic input from Antarctic sources is therefore pivotal to determine the mode of transport of fine fraction material to our sampling locations, in particular because our Nd-Sr isotope data show overlap with the compositions of shelf sediments in the Pacific sector of Antarctica (Fig. 2).

**Fig. 4:**
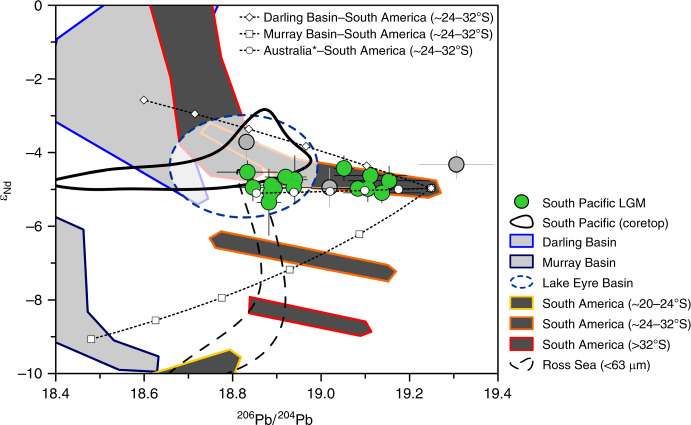
South Pacific Last Glacial Maximum fine fraction samples in Pb–Nd isotope space. Last Glacial Maximum (LGM) data points and error bars represent averages and their 2 SD across the LGM interval (~18–24 ka BP). The South Pacific coretop data include only open ocean locations; samples from the New Zealand and West Antarctic margins^34^, and sample PS75/084-1 are not included (see also Supplementary Note 6). The blue dashed circle approximates the Lake Eyre composition based on the data in Figs. 2 and 3 (see Supplementary Table 3 for references). Mixing between important source regions indicated by stippled lines, white symbols represent 20% increments. Australia* reflects the endmember composition as described in the main text. See Supplementary Table 2 for more details and Supplementary Table 3 for references to literature data. It is noteworthy that South Africa and New Zealand (overlap with Lake Eyre Basin) are not included here (see “Discussion” and Supplementary Note 2 and Supplementary Table 4).

Lithogenic material from Antarctica can reach the mid-latitude South Pacific via the main routes of ice rafting and bottom water export from the Ross Sea^24,42,47,49^. Sediment provenance tracers, which are less sensitive to grain-size effects, such as Nd and Pb isotopes^28,39,42^ (see Supplementary Note 4), support Sr isotope evidence (see Supplementary Note 5) showing that the Ross Sea area was not a major source of < 5 µm fraction sediment supply to our sample locations during the LGM (Figs. 3 and 4). Likewise, northwards penetrating turbidity flows are unable to supply significant amounts of lithogenic fine fraction material to the mid-latitude South Pacific^34^. Accordingly, the zonal diversity of Nd-Sr isotope compositions of West Antarctic shelf sediments^50^ is not reflected in our LGM fine fraction data (Fig. 2). The confined ice-free regions along East Antarctica’s Ross Sea coast were shown to be a significant source of dust to the modern Southwest Ross Sea^24^ and at the nearby Taylor Glacier during the Holocene^48^. However, the dust signal from older ice of the Taylor Glacier was ascribed primarily to input from sources outside of the Antarctic continent^48^. Taking into account that LGM dust fluxes were two orders of magnitude higher in the mid-latitude South Pacific (~120 −200 mg cm^−2^ ka^−1^)^41^ than contemporaneous fluxes at the Taylor Glacier (< ~1.2 mg cm^−2^ ka^−1^)^48^, we consider contributions from Antarctic dust sources to our study area insignificant.

Consequently, the lithogenic < 5 µm sediment fraction was delivered to our South Pacific core locations primarily by airborne transport from terrestrial sources outside of the Antarctic continent, thus supporting previous suggestions that the lithogenic fluxes in the mid-latitude South Pacific reflect predominantly dust input^41,42^. Therefore, our LGM < 5 µm sediment fraction samples are also referred to as dust fraction.

### Two main dust signatures in the glacial South Pacific

The key characteristic of our data set is a distinct latitudinal distribution of Pb isotope compositions of the dust fraction (Fig. 3). This pattern requires mixing of two dominating components including one component with radiogenic Pb isotope compositions and one with less radiogenic Pb isotope compositions dominating in the SAZ and AZ, respectively (Fig. 3b). Importantly, the relatively invariable Nd isotope signatures (*ɛ*_Nd_ of −4.9 ± 0.6, 2 SD, *n* = 70) associated with the two different Pb isotope signals in our samples limit the possible PSA endmember compositions to a narrow range between *ɛ*_Nd_ of ~ −4 and ~ −6 (Fig. 4). This narrow Nd isotope range excludes a possible role for emissions from active volcanoes showing typically highly radiogenic *ɛ*_Nd_ of ~6.5^51^. Moreover, the combined evidence from Pb, Nd, and Sr isotopes excludes South Africa as a significant dust source to our study area, consistent with existing model results^15^ (Figs. 2 and 3).

Australia hosts geochemically diverse source areas^29,32,40^ dominating the dust deposition in the mid-latitude South Pacific in the present day^15,32,34^. The Nd isotope compositions of PSAs in West (*ɛ*_Nd_ of −17 ± 8.3, 2 SD, *n* = 21) and South Australia (*ɛ*_Nd_ of −12.2 ± 6.1, 2 SD, *n* = 38) (Supplementary Table 2)^29,40^ are inconsistent with the observed endmember *ɛ*_Nd_ range between ~ −4 and ~ −6. The large Murray and Darling river basins in East Australia have been considered as important dust source regions during the last glacial period^29,52,53^. The Pb isotope compositions are variable and relatively unradiogenic in the two sub-basins^29,38,54^ (Supplementary Table 2 and Fig. 3b), but systematic Nd isotope differences exist between the Murray (*ɛ*_Nd_ of −9.1 ± 3.4, 2 SD, *n* = 24) and the Darling Basin (*ɛ*_Nd_ of −2.6 ± 4.2, 2 SD, *n* = 19)^29,55^ (Supplementary Table 2 and Fig. 4). Consequently, an integrated signal contributing unradiogenic Pb from the Murray and Darling Basins could be explained by a ~1:1 mixture of sources in the two basins to comply with *ɛ*_Nd_ values of −4.9 ± 0.6 in our LGM dust samples (Fig. 4). However, a 1:1 mixture would imply a large Sr isotope offset between the hypothetical endmember signal (^87^Sr/^86^Sr of 0.7212) (Supplementary Table 2) and our South Pacific dust sample compositions (^87^Sr/^86^Sr of 0.7111 ± 0.0015). Notwithstanding that the Murray–Darling data were obtained from clay fraction sediments (< 2 µm)^55^ (Supplementary Table 3) showing typically elevated ^87^Sr/^86^Sr relative to larger grain sizes^56^ (see also Supplementary Note 4), the magnitude of this offset is difficult to reconcile with documented bias from grain-size effects in dust deposits^28,30,33,56^. Furthermore, it would be surprising if dust flux changes of up to approximately one order of magnitude^52,53^ yielded a relatively stable endmember mixing from geochemically distinct source regions in East Australia during the LGM.

Instead, we suggest that the Lake Eyre Basin in central Australia was the primary source of long-traveled dust carrying unradiogenic Pb to our core sites in the South Pacific during the LGM. A prominent role for this source region has been suggested previously based on Nd and Sr isotope compositions of bulk sediment samples from the central South Pacific^42^. Supporting evidence is provided by the remarkable resemblance of Nd and Sr isotope compositions in the Lake Eyre Basin (*ɛ*_Nd_ of −4.1 ± 2.0 and ^87^Sr/^86^Sr of 0.7112 ± 0.0038, 2 SD, *n* = 17)^29,40^ with < 10 µm fraction samples from the eastern Tasman Sea (*ɛ*_Nd_ of −5.1 ± 1.4 and ^87^Sr/^86^Sr of 0.7115 ± 0.0012, 2 SD, *n* = 2)^40^ (Supplementary Table 2) and our LGM dust samples (Fig. 2). We emphasize that parts of the Darling Basin share geochemical characteristics with the Lake Eyre Basin, which could have contributed to the integrated signal of Australian dust emissions recorded at our core sites in the South Pacific (Figs. 2 and 4).

Similarity exists also between the Nd and Sr isotope compositions of our LGM dust samples (*ɛ*_Nd_ of −4.9 ± 0.6 and ^87^Sr/^86^Sr = 0.7111 ± 0.0015) and < 5 µm fraction sediments from New Zealand’s South Island (*ɛ*_Nd_ of ~ −5.4 ± 3.7, 2 SD, *n* = 10 and ^87^Sr/^86^Sr of 0.7142 ± 0.0103, 2 SD, *n* = 10)^31^ (Supplementary Table 2) (Fig. 2b), which has been considered an important dust source during glacial periods^16,20,41,42^. Based on Nd, Sr, and Pb isotope compositions, it is not possible to unambiguously differentiate between the integrated Australian dust signal and New Zealand PSAs (Supplementary Table 2). Complementary REE data conflict with an important role of dust supply from New Zealand to our study area (Supplementary Note 2 and Supplementary Fig. 3). We note that the published dust fraction REE data from New Zealand are very limited and may not reflect the full range of New Zealand PSA REE compositions (Supplementary Fig. 3). Based on the currently available evidence, we use central Australian Pb^38^ and Tasman Sea Nd isotope data^40^ to constrain the composition of the dust endmember signal exported from Australia during the LGM (Supplementary Table 2). This central Australian source can explain up to 100% of the total dust deposition in the South Pacific AZ (Fig. 4).

The second component dominating in the South Pacific SAZ requires a dust source with radiogenic Pb isotope compositions of ^206^Pb/^204^Pb > 19, which are not characteristic for Australian PSAs^38,54^ (Fig. 3b and Supplementary Table 2). The dust fraction of LGM sediments from the New Zealand margin yielded ^206^Pb/^204^Pb of 19.31 ± 0.09 (2 SD, *n* = 5), which could represent a possible source of radiogenic Pb to the South Pacific SAZ (Fig. 3). However, these sediments are distinct from the PSA signatures on land and reflect a local signal limited to the New Zealand continental shelf and margin environment (see Supplementary Note 3), probably related to mineral sorting during riverine, coastal, and/or submarine sediment transport^57^. Terrestrial PSAs with sufficiently radiogenic Pb isotope compositions that could account for the second endmember in our Pb isotope array are rare in the Southern Hemisphere. South America is often considered the major source of long-traveled dust in the Southern Hemisphere during glacial periods^28,31,38,39^. Many South American PSAs, such as the Puna-Altiplano region between ~20° and 24°S and PSAs south of ~32° including Patagonia (>38°S), show geochemical fingerprints that are inconsistent with the two-component mixing that is evident from our South Pacific dust fraction samples (Figs. 3 and 4).

From the main Southern Hemisphere PSA, only the area between ~24°S and 32°S in Central South America hosts deposits with Pb and Nd isotope compositions that can account for the second endmember dominating the dust fraction in the South Pacific SAZ (Figs. 3 and 4, and Supplementary Fig. 4). The Pb and Nd isotope characteristics of two < 63 µm fraction samples from ~26°S (^206^Pb/^204^Pb of 19.15 and *ɛ*_Nd_ of ~ −7) and ~28°S (^206^Pb/^204^Pb of 19.25 and *ɛ*_Nd_ of ~ −5)^28,39^ are consistent with the composition of (volcano-)sedimentary rock deposits in the area reflecting a mixed signal of mantle-derived material and older continental crust^33,58,59^. The possible endmember Pb and Nd isotope compositions (^206^Pb/^204^Pb of 19.25 and *ɛ*_Nd_ of ~ −5) feature relatively radiogenic ^87^Sr/^86^Sr of 0.7169 in the < 5 µm sediment fraction^28^ (Supplementary Table 2), which seems inconsistent with the composition of our South Pacific dust samples (Fig. 2b). However, this can be reconciled with changes in the PSA weathering regime and/or different proportions of clay minerals in dust deposits relative to their source soils (see Supplementary Note 4). We suggest that the entrainment, long-range transport and/or deposition of dust discriminated against clay minerals with typically radiogenic Sr isotope compositions (high ^87^Sr/^86^Sr)^33,56^, thus inducing a bias towards lower ^87^Sr/^86^Sr at the site of deposition. For example, particle size distributions of Central South American dust trap and source soil samples indicate a depletion of clay-sized particles in airborne dust relative to the source soils^60^. Such depletion may be related to the emission of clay minerals in aggregates and their preferential removal during transport relative to smaller, fully dispersed clay minerals^43,61^. Supporting evidence for this hypothesis is provided by the low abundance or absence of the clay minerals smectite and kaolinite in East Antarctic ice cores^44,62^, which are important components of soils in the PSAs of South America and Australia^63,64^.

Using the most radiogenic Pb isotope signatures from Central South America (Supplementary Table 2), mixing calculations reveal that Central South America contributed up to ~80% to the glacial dust deposition in the South Pacific SAZ, whereas the South Pacific AZ received up to 100% of dust from central Australia (Figs. 3b and 4). This finding is surprising, because Patagonia (south of 38°S) is typically considered the major source of far-traveled dust coming from South America^6,31,37,38^, although Central South America has been invoked as an important dust source to the South Atlantic and East Antarctica^28,33,39^. The pattern is also fundamentally different to the modern situation that is characterized by Australian dust dominating in the entire study area^15,34^ and provides specific information about entrainment, transport, and deposition of airborne mineral dust during the LGM.

### Glacial dust entrainment and circumpolar transport

We show that dust in the LGM South Pacific AZ was primarily derived from Australia. High emissions from central Australian dust sources are consistent with previous work on modern and past environments^29,35,40,52,53,65,66^. The combination of river systems supplying sediments to extensive alluvial plains and ephemeral lake plays could support efficient deflation of fine material in the expanded (semi)arid zone of central Australia during the LGM^29,53^. Australian dust activity typically follows a seasonal cycle peaking during the spring and summer seasons, as a result of the complex interaction of migrating atmospheric pressure systems with sediment availability and erosivity in the dust source areas^26,27,29,53^. In analogy to the modern situation, the distribution of mineral dust from Australia across the Tasman Sea^32,40^ and the South Pacific was orchestrated by the eastward moving frontal systems of the SWW belt during the LGM.

The situation in the South Pacific SAZ is different. The prominent role of Central South America as a major dust source to the South Pacific SAZ during the LGM has not previously been shown. Modern observations and model simulations show pronounced dust activity in Central South American PSAs near ~30°S^27,60,67^. Importantly, Central South American PSAs feature a number of environmental preconditions for the efficient production of fine particles including high relief, (semi)arid conditions with intermittent fluvial activity^26,27,33,67,68^, and increased glacier activity during glacial intervals^69,70^.

One mechanism to transport dust from South America to the South Pacific is westward by the trade winds. However, our study area is south of the low-latitude trade wind system today (Supplementary Fig. 1) and the Pb isotope composition of dust transported by the (sub)tropical Pacific trade winds during the LGM (^206^Pb/^204^Pb of 18.69 ± 0.05, 2 SD, *n* = 7 including replicates)^71^ is inconsistent with the dust signal recorded in the South Pacific SAZ (^206^Pb/^204^Pb of 19.09 ± 0.17, 2 SD, *n* = 28) (Fig. 3). Therefore, the only viable route of Central South American dust to the South Pacific SAZ was eastward on a circum-Antarctic path within the mid-latitude SWW belt (Supplementary Figs. 1 and 9).

**Fig. 5:**
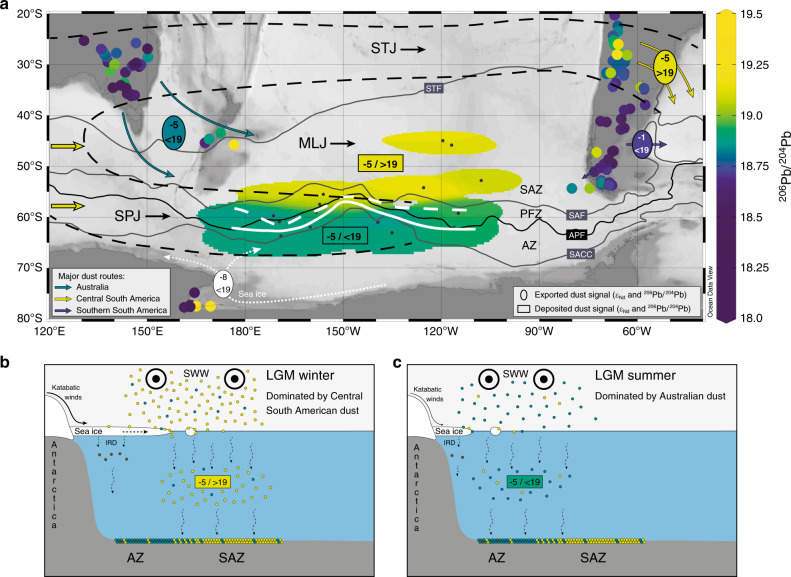
The transport and deposition of dust in the South Pacific during the Last Glacial Maximum. **a** The distribution of dust fraction ^206^Pb/^204^Pb in the glacial South Pacific. Solid and dashed white lines delineate the limits of 40% and 15% winter sea-ice cover during the Last Glacial Maximum (LGM)^77^, respectively. Black dots: LGM time slice sampling locations. The dashed black outline indicates the modern zones of increased wintertime wind speed maxima in the high-altitude (200 hPa) westerly wind flow of the subtropical (STJ) and subpolar (SPJ) branches of the jet stream located north and south of the reduced mid-latitude jet (MLJ), respectively^73^. Terrestrial source data from the literature listed in Supplementary Table 3. It is noteworthy that some original terrestrial sample coordinates were slightly modified to improve visibility in this figure. Ocean fronts from ref. ^94^. STF: Subtropical Front. SAF: Subantarctic Front. APF: Antarctic Polar Front. SACC: Southern ACC Front. SAZ: Subantarctic Zone. PFZ: Polar Frontal Zone. AZ: Antarctic Zone. Map created with Ocean Data View software^95^. **b** The LGM wintertime scenario. The northward expanded Southern Hemisphere westerly wind (SWW) system (STJ present in the South Pacific as in **a**) delivers dust efficiently on a circumpolar trajectory from Central South America to the study area. The extensive sea-ice cover reduces the dust deposition in the South Pacific AZ. IRD: ice-rafted detritus. Numbers in yellow box as in **a**. **c** The LGM summertime scenario. The STJ transporting dust from Central South America is absent, the SWW move closer to Antarctica and deliver predominantly Australian dust to the South Pacific AZ and SAZ north of the summer sea-ice limit. Numbers in green box as in **a**. A comprehensive overview of tracer evidence constraining our proposed LGM scenario of circumpolar dust transport and deposition in the study area is provided in Supplementary Table 4.

Here we propose that increased SWW activity in Central South America north of ~30°S facilitated efficient dust entrainment during the LGM, in particular during the winter season when the SWW regime reaches its northernmost extension^68,72^. In the present day, the mid-latitude SWW regime intersecting South America spans the lower troposphere westerly winds and the high-altitude jet streams^33,72–74^. The westerly winds are typically associated with strong dry foehn-like winds promoting dust entrainment on the eastern side of the Andes^28,30,33,39,60,67,75^. The high-altitude westerly jet circulation features a mid-latitude jet stream in the Southern Hemisphere, which is characterized by a weakened subpolar (~60°S) and a strengthened subtropical branch (~30°S) over the South Pacific during austral winter^73^ (Fig. 5a). Modern observations, trajectory modeling, and geochemical evidence show that the subtropical jet stream plays an important role in the export and long-range transport of dust from high-altitude PSAs in Central South America^33,60^ (Supplementary Fig. 9). Recent work suggested that the subtropical jet stream was instrumental to enhance fluvial input at the Chilean continental slope near 27.5°S off the southern Atacama Desert during past glacials^68^.

We therefore conclude that SWW-induced orographic winds and intensified subtropical jet stream circulation^28,33,39,60,68,72^, possibly augmented by enhanced availability of fine material from glacier and (ephemeral) fluvial activity^33,68–70^, enabled efficient dust entrainment in and export from Central South American PSAs during the LGM. This scenario is consistent with the provenance of Argentinean loess and dust samples collected downwind of our suggested source region^28,30,39,60,75^, and with independent provenance constraints derived from helium‒thorium isotope compositions of sediments in the South Atlantic^76^. The long-distance circumpolar transport of dust (Supplementary Fig. 9) was likely promoted by reduced wet deposition and increased lifetime of atmospheric dust during the LGM^15^. The increased abundance and prolonged residence time of < 5 µm particles from Central South America would have enhanced scattering of incoming solar radiation, thus contributing directly to glacial cooling^43^. Different from the modern situation^34^ and LGM model simulations^15^, these boundary conditions allowed Central South American dust to outpace the deposition from Australian sources in the South Pacific SAZ during the LGM (Fig. 5a and Supplementary Table 4). The interplay of these two important terrestrial PSAs may explain the increased Pb isotope variability in particular in the South Pacific SAZ during the LGM interval (Fig. 3) and could have been involved in the geochemical variability of dust samples from Antarctic ice cores^28,31,33,38,39,44,48^.

### Dust deposition in the glacial South Pacific

The two distinct dust depositional environments in the LGM South Pacific are characterized by a narrow transition zone (Fig. 5a), a pattern that cannot be controlled by variable atmospheric dust transport alone. Therefore, we invoke local oceanic processes modulating the settling of atmospheric dust fallout to the seafloor. The delineation between the two dust depositional zones shows remarkable correspondence with the position of the modern polar front and the LGM winter sea-ice cover^77^ (Fig. 5a). A primary control by hydrodynamic processes seems unlikely given the lacking correspondence between dust fraction geochemistry and the ACC fronts in the present day^34^. A characteristic feature of Southern Ocean sea-ice cover is a pronounced seasonal cycle^77^ such that the dust deposition at locations under the LGM winter sea-ice cover was reduced when dust transport from Central South American PSAs was presumably most efficient due to the enhanced northward expansion of the SWW/jet stream regime during the LGM winter season (see above). The offshore motion of wind and sea ice^24,49^ would move the wintertime South American dust fallout out of the ice-covered ocean areas of the South Pacific AZ (Fig. 5a, b). A sea-ice control on the deposition of lithogenic particles is also evident from modern sediment trap data showing reduced lithogenic fluxes in the South Pacific AZ and increased fluxes in the SAZ^78,79^. However, it may be speculated if additional processes were involved in the concentration of wintertime dust in the South Pacific SAZ during the LGM. For example, settling of particles is typically enhanced by aggregation^80,81^ explaining also the close correspondence of biogenic and lithogenic particle fluxes in the Southern Ocean^79^. Dust particles released from sea ice to the AZ surface ocean during the melting season^23,24^ would be subject to further north(east)ward dispersal by the surface currents^49^. The earlier resumption of the primary productivity in the ice-free SAZ in spring^78,79^ could enhance the transfer of wintertime dust to the seafloor. During the rest of the year, the SWW/jet stream regime would not provide an efficient dust conveyor from Central South America to the South Pacific. Then, Australian dust fallout could dominate over the entire study area, including the region that is seasonally ice-free, but without outpacing the (winter) input from Central South America in the South Pacific SAZ (Fig. 5c).

As a result, the South Pacific SAZ was dominated by highly seasonal dust input from Central South American sources accounting for ~50‒80% of the total deposition and hence, for large parts of the threefold increase in glacial South Pacific SAZ dust fluxes^41^. We suggest that the seasonality of atmospheric dust supply from Central South America and sea-ice cover played important roles in modulating particle settling in the glacial South Pacific, thus inducing the distinct north–south trend in dust provenance evident from our data set (Fig. 5).

### Dust provenance changes and Southern Ocean Fe supply

Previous estimates of a dust-induced ~40 p.p.m. lowering of glacial atmospheric CO_2_ concentrations critically rely on reconstructions from the South Atlantic SAZ, extrapolation of the results to the entire Southern Ocean, and a uniform value of 2% Fe solubility from mineral dust fallout^5,6^. However, the distinct pattern of LGM South Pacific dust provenance implies a change in source area particle mineralogy and in dust transport conditions, both of which can directly affect dust-Fe solubility^17,18,20,21^ with possible cascading effects on biogeochemical reactions in the atmosphere and in seawater^18,22,25^. Recent laboratory experiments with natural dust samples showed a close relationship between diatom growth and the content of more soluble Fe(II)-rich silicate minerals typical for glaciogenic dust sources^19^. Reconstructions estimate a relatively uniform ~15-fold increase of Fe(II) supply to the glacial Southern Ocean in comparison to interglacial periods^20^. The similar phasing and magnitude of the glacial Fe(II) increase in the South Pacific and South Atlantic was related to the synchronized activity of chemically pristine (low degree of chemical weathering) glaciogenic mineral dust emissions from New Zealand and Patagonia, respectively^20^. Non-glaciogenic Australian PSA sediments show variable degrees of chemical maturity^64^, but dust from these sources might host less soluble Fe than chemically more pristine dust^17,21^. The contribution of Australian dust to the South Pacific could reduce the efficiency of a dust-induced glacial drawdown of atmospheric CO_2_ to less than the proposed ~40 p.p.m. Yet, our new data set suggests that the increased supply of primary Fe(II) silicates to the South Pacific SAZ^20^ was largely driven by mineral dust from chemically more pristine sources in (semi)arid Central South America^28,39,59,82^ (Figs. 4 and 5a). The solubility of dust-borne Fe from Central South America was probably further increased during the long circum-Antarctic transport^22^ and by (winter) sea-ice processing in the study area^23^. A possible reduction of Fe(II) supply by the contribution of more mature Australian mineral dust is then rendered negligible by the large-scale deposition and sea-ice processing of long-traveled Central South American dust. As such, our data can explain the zonally symmetric increase in Fe(II) supply to the Southern Ocean SAZ^20^, which stimulated primary productivity^19^, nutrient consumption^6,13^, and export production^5^, thus supporting the increased sequestration of carbon in the glacial deep ocean^4,14^.

As a corollary, dust-borne Fe input as a critical component of the glacial–interglacial Fe feedback in the Southern Ocean was characterized by the interaction of the SWW system with changes in specific Central South American PSAs. This implies that Southern Ocean dust-Fe fertilization is highly sensitive to the environmental conditions in this important dust source region. Our findings are consistent with recent reconstructions of Fe supply to the subpolar Southern Ocean, but revise previous hypotheses of the source of the dust-borne Fe supply^5,6,20,41,42^. It may be speculated whether the enhanced dust emissions from these Central South American PSAs were systematic and linear during the past glacial–interglacial cycles or if there was threshold behavior. The precessional forcing of the South Pacific subtropical jet stream dynamics^68^ would suggest that the LGM may not reflect whole glacial averages. Rather, our proposed link between dust activity in the source regions, the variable SWW system and the input of dust-borne Fe to the South Pacific SAZ indicates that the Southern Hemisphere dust-Fe supply can operate as a dynamic feedback on multiple time scales.

## Methods

### Grain-size separation and sediment leaching

For this study, we sampled about three cubic centimeters of wet bulk sediment for wet-sieving and subsequent Stokes-based separation of the < 5 µm grain-size fraction in glass settling tubes at the Alfred Wegener Institute Bremerhaven. The < 5 µm fraction was then subjected to a sequential leaching procedure at the Institute for Chemistry and Biology of the Marine Environment (ICBM) in Oldenburg to extract the lithogenic silicate fraction^34^ (see also Supplementary Note 1 for more details). The < 5 µm fraction samples were processed in a total of eight batches. One batch contained typically twelve samples, two procedural blanks, and the two United States Geological Survey (USGS) rock reference materials AGV-1 and BCR-2. All chemical and analytical sample processing was carried out at the ICBM using high-purity reagents^34^ if not indicated otherwise.

An aqueous solution with ~5% H_2_O_2_ was used to eliminate organics, followed by a two-step 1 M HCl leach (3 h and overnight) to remove carbonates and ferromanganese (oxy-)hydroxides. During a final step, the samples were exposed to 0.03 M EDTA (99.995% trace metals basis, Sigma-Aldrich^®^) to sequester any remaining free and loosely adsorbed metal ions. After all steps the supernatant reagent was removed and the samples were triple-rinsed with Milli-Q^®^ H_2_O. Then, the samples were freeze-dried and ~50–100 mg (depending on opal content) of freeze-dried sample material was weighed into ultra-clean polytetrafluoroethylene (PTFE) vessels fitting the PicoTrace DAS 30 pressure digestion system. To break down any remaining organic compounds, the samples were exposed to aqua regia at 130 °C overnight, followed by pressure digestion at a nominal temperature of 230 °C (180 °C measured in the digestion unit) using a mixture of concentrated HF-HNO_3_-HClO_4_. After complete digestion, the samples were converted to chloride form, redissolved in 6 M HCl, and split for REE analyses (see Supplementary Methods) and sequential wet-chemical extraction of target elements from the sample matrix following previously published procedures^34^. In brief, the sample aliquot was converted to bromide form for HBr-HNO_3_-based Pb extraction using Biorad^®^ AG1-X8 resin^83^. The matrix wash fraction was collected to separate the alkaline earth metals from the REE using Biorad^®^ AG50W X-8 resin^84^. Strontium was extracted from the remaining sample matrix using Eichrom Sr resin^85^ and Nd was isolated from the LREE with TrisKem Ln resin-based chemistry^86^.

### Radiogenic isotope analyses

The Pb, Nd, and Sr isotope compositions were determined using a Thermo Scientific^TM^ Neptune Plus^TM^ multi-collector ICP-MS at the ICBM in Oldenburg. All reported AGV-1 and BCR-2 reference material results were obtained on leached residues (see above). For Nd isotope analyses, mass bias was corrected for using ^146^Nd/^144^Nd = 0.7219 and an exponential law. Isobaric interferences of ^142^Ce and ^144^Sm on ^142^Nd and ^144^Nd were monitored and corrected for using ^140^Ce and ^147^Sm, respectively. The JNdi-1 reference material was measured every four samples to correct for the instrumental offset of the mass bias corrected ^143^Nd/^144^Nd ratios of the samples to JNdi-1 reference ratio of 0.512115 ± 0.000007^87^. The external reproducibility of normalized ^143^Nd/^144^Nd ratios of acid-leached BCR-2 and AGV-1 rock powders was 0.512641 ± 0.000013 (2 SD, *n* = 29) and 0.512794 ± 0.000020 (2 SD, *n* = 32), respectively. These results are in excellent agreement with literature ^143^Nd/^144^Nd of 0.512637 ± 0.000013 for acid-leached BCR-2 residues^88^ and 0.512791 ± 0.000013 for unleached AGV-1 rock powder^89^. Repeat analyses of sample C7 yielded ^143^Nd/^144^Nd = 0.512387 ± 0.000011 (2 SD, *n* = 16). All sample Nd isotope results are expressed in epsilon notation: *ε*_Nd_ = (^143^Nd/^144^Nd_sample_)/(^143^Nd/^144^Nd_CHUR_) − 1] × 10^4^ where CHUR is the Chondritic Uniform Reservoir^90^.

For Sr isotope analyses, mass bias was corrected for using ^86^Sr/^88^Sr = 0.1194 and an exponential law. Krypton gas blanks, measured on ^83^Kr to correct for ^86^Kr on ^86^Sr, were below 0.1 mV, whereas ^86^Sr was measured at 2‒3 V. Isobaric interferences from ^87^Rb on ^87^Sr were monitored and corrected using ^85^Rb. Repeat analyses of NIST SRM987 (every four samples) were used to normalize sample ^87^Sr/^86^Sr to SRM987 ^87^Sr/^86^Sr of 0.710248^91^. The external reproducibility of acid-leached BCR-2 and AGV-1 was ^87^Sr/^86^Sr = 0.704994 ± 0.000027 (2 SD, *n* = 32) and 0.703950 ± 0.000025 (2 SD, *n* = 32), respectively. These values are indistinguishable from the values reported previously for acid-leached residues of BCR-2 (0.705000 ± 0.000011)^88^ and AGV-1 reference materials (0.703950 ± 0.000012)^89^. Repeat analyses of sample C7 yielded Sr/^86^Sr = 0.710887 ± 0.000021 (2 SD, *n* = 12).

Measurements of the Pb isotope compositions were performed as standard-sample bracketing using NIST SRM981 as bracketing standard^92^. Isobaric interference of ^204^Hg on ^204^Pb was corrected for through monitoring ^202^Hg (< 0.5 mV), whereas ^204^Pb was typically measured at ~300‒400 mV. The instrumental mass bias of sample analyses was corrected for by normalization of the measured SRM981 values to the accepted values of ref. ^93^. The external reproducibility was monitored through repeat analyses of secondary reference materials BCR-2 and AGV-1 (literature values in brackets). Selected Pb isotope results for BCR-2 (2 SD, *n* = 39) were ^206^Pb/^204^Pb = 18.8030 ± 0.0025 (18.8029 ± 0.0010)^88^, ^207^Pb/^204^Pb = 15.6253 ± 0.0019 (15.6239 ± 0.0008)^88^, and ^208^Pb/^204^Pb = 38.8337 ± 0.0065 (38.8287 ± 0.0025)^88^. Repeat analyses of AGV-1 (2 SD, *n* = 39) yielded ^206^Pb/^204^Pb = 18.9034 ± 0.0026 (18.9054 ± 0.0013)^89^, ^207^Pb/^204^Pb = 15.6147 ± 0.0016 (15.6165 ± 0.0001)^89^, and ^208^Pb/^204^Pb = 38.5737 ± 0.0071 (38.5875 ± 0.0220)^89^ for the same analytical sessions. Our Pb isotope results on leached BCR-2 and AGV-1 rock powders demonstrate excellent reproducibility of existing literature values^88,89^ and previous results from the ICBM lab^34^. Repeat analyses of sample C7 (*n* = 7) yielded ^206^Pb/^204^Pb = 19.1504 ± 0.0017, ^207^Pb/^204^Pb = 15.6657 ± 0.0015, and ^208^Pb/^204^Pb = 39.1677 ± 0.0042. It is noted that our results on acid-leached BCR-2 and AGV-1 rock powders are compared to the acid-leached literature results where available, and that the literature Pb isotope ratios of acid-leached AGV-1 residues are based on only two analyses^89^. The procedural blanks for Nd, Pb, and Sr were below 40 pg, 120 pg, and 2.5 ng, respectively. Typically, the blank contaminations were significantly less than 1% of the individual sample Nd, Pb, and Sr yields with negligible effects on the respective sample isotope compositions (see also Supplementary Note 6 for more details).

## Supplementary information

Supplementary Information

Supplementary Data 1

Supplementary Data 2

Supplementary Data 3

Description of additional supplementary files

## Data Availability

All data presented in this paper are included in this published article and its Supplementary Data files.

## References

[1] Lüthi D (2008). High-resolution carbon dioxide concentration record 650,000–800,000 years before present. Nature.

[2] Lambert F (2008). Dust-climate couplings over the past 800,000 years from the EPICA Dome C ice core. Nature.

[3] Parrenin F (2013). Synchronous change of atmospheric CO2 and antarctic temperature during the last deglacial warming. Science.

[4] Sigman DM, Boyle EA (2000). Glacial/interglacial variations in atmospheric carbon dioxide. Nature.

[5] Martinez-Garcia A (2011). Southern Ocean dust-climate coupling over the past four million years. Nature.

[6] Martínez-García A (2014). Iron fertilization of the subantarctic ocean during the last ice age. Science.

[7] Jaccard SL (2013). Two modes of change in Southern Ocean productivity over the past million years. Science.

[8] Martin JH (1990). Glacial-interglacial CO_2_ change: the iron hypothesis. Paleoceanography.

[9] Boyd PW (2007). Mesoscale iron enrichment experiments 1993–2005: synthesis and future directions. Science.

[10] Pollard RT (2009). Southern Ocean deep-water carbon export enhanced by natural iron fertilization. Nature.

[11] Boyd PW, Claustre H, Levy M, Siegel DA, Weber T (2019). Multi-faceted particle pumps drive carbon sequestration in the ocean. Nature.

[12] Yoon J-E (2018). Reviews and syntheses: ocean iron fertilization experiments – past, present, and future looking to a future Korean Iron Fertilization Experiment in the Southern Ocean (KIFES) project. Biogeosciences.

[13] Wang XT (2017). Deep-sea coral evidence for lower Southern Ocean surface nitrate concentrations during the last ice age. Proc. Natl Acad. Sci. USA.

[14] Khatiwala S, Schmittner A, Muglia J (2019). Air-sea disequilibrium enhances ocean carbon storage during glacial periods. Sci. Adv..

[15] Albani S, Mahowald NM, Delmonte B, Maggi V, Winckler G (2012). Comparing modeled and observed changes in mineral dust transport and deposition to Antarctica between the Last Glacial Maximum and current climates. Clim. Dyn..

[16] Neff PD, Bertler NAN (2015). Trajectory modeling of modern dust transport to the Southern Ocean and Antarctica. J. Geophys. Res. Atmos..

[17] Schroth AW, Crusius J, Sholkovitz ER, Bostick BC (2009). Iron solubility driven by speciation in dust sources to the ocean. Nat. Geosci..

[18] Shi Z (2012). Impacts on iron solubility in the mineral dust by processes in the source region and the atmosphere: a review. Aeolian Res..

[19] Shoenfelt EM (2017). High particulate iron(II) content in glacially sourced dusts enhances productivity of a model diatom. Sci. Adv..

[20] Shoenfelt EM, Winckler G, Lamy F, Anderson RF, Bostick BC (2018). Highly bioavailable dust-borne iron delivered to the Southern Ocean during glacial periods. Proc. Natl Acad. Sci. USA.

[21] Mackie, D. S. et al. Biogeochemistry of iron in Australian dust: from eolian uplift to marine uptake. *Geochem. Geophys. Geosyst*. **9** (2008).

[22] Baker AR, Croot PL (2010). Atmospheric and marine controls on aerosol iron solubility in seawater. Mar. Chem..

[23] Albani S (2016). Paleodust variability since the Last Glacial Maximum and implications for iron inputs to the ocean. Geophys. Res. Lett..

[24] Winton VHL (2016). The origin of lithogenic sediment in the south-western Ross Sea and implications for iron fertilization. Ant. Sci..

[25] Shaked Y, Buck KN, Mellett T, Maldonado MT (2020). Insights into the bioavailability of oceanic dissolved Fe from phytoplankton uptake kinetics. ISME J..

[26] Prospero JM, Ginoux P, Torres O, Nicholson SE, Gill TE (2002). Environmental characterization of global sources of atmospheric soil dust identified with the Nimbus 7 total ozone mapping spectrometer (toms) absorbing aerosol product. Rev. Geophys..

[27] Ginoux P, Prospero JM, Gill TE, Hsu NC, Zhao M (2012). Global-scale attribution of anthropogenic and natural dust sources and their emission rates based on MODIS Deep Blue aerosol products. Rev. Geophys..

[28] Gili S (2017). Glacial/interglacial changes of Southern Hemisphere wind circulation from the geochemistry of South American dust. Earth Planet. Sci. Lett..

[29] De Deckker P (2019). An evaluation of Australia as a major source of dust. Earth Sci. Rev..

[30] Smith J (2003). Isotopic constraints on the source of Argentinian loess – with implications for atmospheric circulation and the provenance of Antarctic dust during recent glacial maxima. Earth Planet. Sci. Lett..

[31] Delmonte B (2004). Comparing the Epica and Vostok dust records during the last 220,000 years: stratigraphical correlation and provenance in glacial periods. Earth Sci. Rev..

[32] McGowan HA, Kamber B, McTainsh GH, Marx SK (2005). High resolution provenancing of long travelled dust deposited on the Southern Alps, New Zealand. Geomorphology.

[33] Gaiero, D. M. Dust provenance in Antarctic ice during glacial periods: from where in southern South America? *Geophys. Res. Lett*. **34** (2007).

[34] Wengler M (2019). A geochemical approach to reconstruct modern dust fluxes and sources to the South Pacific. Geochim. Cosmochim. Acta.

[35] De Deckker P (2020). Airborne dust traffic from Australia in modern and Late Quaternary times. Glob. Planet. Change.

[36] Grousset FE (1992). Antarctic (Dome C) ice-core dust at 18 k.y. B.P.: isotopic constraints on origins. Earth Planet. Sci. Lett..

[37] Delmonte B (2010). Aeolian dust in the Talos Dome ice core (East Antarctica, Pacific/Ross Sea sector): Victoria Land versus remote sources over the last two climate cycles. J. Quat. Sci..

[38] Vallelonga P (2010). Lead isotopic compositions in the EPICA Dome C ice core and Southern Hemisphere potential source areas. Quat. Sci. Rev..

[39] Gili S (2016). Provenance of dust to Antarctica: a lead isotopic perspective. Geophys. Res. Lett..

[40] Revel-Rolland M (2006). Eastern Australia: a possible source of dust in East Antarctica interglacial ice. Earth Planet. Sci. Lett..

[41] Lamy F (2014). Increased dust deposition in the Pacific Southern Ocean during glacial periods. Science.

[42] Molina‐Kescher M (2016). Reduced admixture of North Atlantic Deep Water to the deep central South Pacific during the last two glacial periods. Paleoceanography.

[43] Kok JF (2017). Smaller desert dust cooling effect estimated from analysis of dust size and abundance. Nat. Geosci..

[44] Delmonte B (2017). Causes of dust size variability in central East Antarctica (Dome B): atmospheric transport from expanded South American sources during Marine Isotope Stage 2. Quat. Sci. Rev..

[45] Wengler, M. Dust variability and provenance in the Pacific and Atlantic sectors of the Southern Ocean. PhD thesis (Bremen, 2018).

[46] McCave IN, Manighetti B, Robinson SG (1995). Sortable silt and fine sediment size/composition slicing: parameters for palaeocurrent speed and palaeoceanography. Paleoceanography.

[47] Hemming SR (2007). Strontium isotope tracing of terrigenous sediment dispersal in the Antarctic Circumpolar Current: implications for constraining frontal positions. Geochem. Geophys. Geosyst..

[48] Aarons SM (2017). Dust composition changes from Taylor Glacier (East Antarctica) during the last glacial-interglacial transition: a multi-proxy approach. Quat. Sci. Rev..

[49] Holland PR, Kwok R (2012). Wind-driven trends in Antarctic sea-ice drift. Nat. Geosci..

[50] Simões Pereira P (2018). Geochemical fingerprints of glacially eroded bedrock from West Antarctica: detrital thermochronology, radiogenic isotope systematics and trace element geochemistry in Late Holocene glacial-marine sediments. Earth Sci. Rev..

[51] Basile I, Petit JR, Touron S, Grousset FE, Barkov N (2001). Volcanic layers in Antarctic (Vostok) ice cores: source identification and atmospheric implications. J. Geophys. Res..

[52] Petherick LM, McGowan HA, Kamber BS (2009). Reconstructing transport pathways for late Quaternary dust from eastern Australia using the composition of trace elements of long traveled dusts. Geomorphology.

[53] Fitzsimmons KE (2013). Late Quaternary palaeoenvironmental change in the Australian drylands. Quat. Sci. Rev..

[54] De Deckker P, Norman M, Goodwin ID, Wain A, Gingele FX (2010). Lead isotopic evidence for an Australian source of aeolian dust to Antarctica at times over the last 170,000 years. Palaeogeogr. Palaeoclimatol. Palaeoecol..

[55] Gingele FX, Deckker PD (2005). Clay mineral, geochemical and Sr–Nd isotopic fingerprinting of sediments in the Murray–Darling fluvial system, southeast Australia. Aust. J. Earth Sci..

[56] Feng J-L, Zhu L-P, Zhen X-L, Hu Z-G (2009). Grain size effect on Sr and Nd isotopic compositions in eolian dust: implications for tracing dust provenance and Nd model age. Geochem. J..

[57] Garçon M, Chauvel C, France-Lanord C, Limonta M, Garzanti E (2014). Which minerals control the Nd–Hf–Sr–Pb isotopic compositions of river sediments?. Chem. Geol..

[58] Lucassen F (2001). Composition and density model of the continental crust at an active continental margin—the Central Andes between 21° and 27°S. Tectonophysics.

[59] Bock B, Bahlburg H, Wörner G, Zimmermann U (2000). Tracing crustal evolution in the southern Central Andes from Late Precambrian to Permian with geochemical and Nd and Pb isotope data. J. Geol..

[60] Gaiero DM (2013). Ground/satellite observations and atmospheric modeling of dust storms originating in the high Puna-Altiplano deserts (South America): implications for the interpretation of paleo-climatic archives. J. Geophys. Res. Atmos..

[61] Perlwitz JP, Pérez García-Pando C, Miller RL (2015). Predicting the mineral composition of dust aerosols – Part 1: representing key processes. Atmos. Chem. Phys..

[62] Gaudichet A (1988). Mineralogy of insoluble particles in the Vostok Antarctic ice core over the last climatic cycle (150 kyr). Geophys. Res. Lett..

[63] Gaiero DM, Depetris PJ, Probst J-L, Bidart SM, Leleyter L (2004). The signature of river- and wind-borne materials exported from Patagonia to the southern latitudes: a view from REEs and implications for paleoclimatic interpretations. Earth Planet. Sci. Lett..

[64] Gingele FX, De Deckker P, Hillenbrand C-D (2004). Late Quaternary terrigenous sediments from the Murray Canyons area, offshore South Australia and their implications for sea level change, palaeoclimate and palaeodrainage of the Murray–Darling Basin. Mar. Geol..

[65] Marx SK, Kamber BS, McGowan HA (2005). Provenance of long-travelled dust determined with ultra-trace-element composition: a pilot study with samples from New Zealand glaciers. Earth Surf. Process. Landf..

[66] Marx SK, McGowan HA, Kamber BS (2009). Long-range dust transport from eastern Australia: a proxy for Holocene aridity and ENSO-type climate variability. Earth Planet. Sci. Lett..

[67] Cosentino NJ (2020). Atmospheric dust dynamics in southern South America: a 14-year modern dust record in the loessic Pampean region. Holocene.

[68] Lamy F (2019). Precession modulation of the South Pacific westerly wind belt over the past million years. Proc. Natl Acad. Sci. USA.

[69] Ammann C, Jenny B, Kammer K, Messerli B (2001). Late Quaternary glacier response to humidity changes in the arid Andes of Chile (18–29°S). Palaeogeogr. Palaeoclimatol. Palaeoecol..

[70] Zech J, Terrizzano C, García-Morabito E, Veit H, Zech R (2017). Timing and extent of late pleistocene glaciation in the arid Central Andes of Argentina and Chile (22°-41°S). Cuad. Investig. Geogr..

[71] Pichat S, Abouchami W, Galer SJG (2014). Lead isotopes in the Eastern Equatorial Pacific record Quaternary migration of the South Westerlies. Earth Planet. Sci. Lett..

[72] Chiang JCH, Lee S-Y, Putnam AE, Wang X (2014). South Pacific Split Jet, ITCZ shifts, and atmospheric North–South linkages during abrupt climate changes of the last glacial period. Earth Planet. Sci. Lett..

[73] Bals-Elsholz TM (2001). The Wintertime Southern Hemisphere Split Jet: structure, variability, and evolution. J. Clim..

[74] Garreaud R, Lopez P, Minvielle M, Rojas M (2013). Large-scale control on the Patagonian climate. J. Clim..

[75] Milana JP, Kröhling DM (2017). First data on volume and type of deflated sediment from Southern Puna Plateau and its role as source of the Chaco-Pampean loess. Quat. Int..

[76] McGee D (2016). Tracking eolian dust with helium and thorium: impacts of grain size and provenance. Geochim. Cosmochim. Acta.

[77] Benz V, Esper O, Gersonde R, Lamy F, Tiedemann R (2016). Last Glacial Maximum sea surface temperature and sea-ice extent in the Pacific sector of the Southern Ocean. Quat. Sci. Rev..

[78] Honjo S, Francois R, Manganini S, Dymond J, Collier R (2000). Particle fluxes to the interior of the Southern Ocean in the Western Pacific sector along 170°W. Deep Sea Res. Part II Top. Stud. Oceanogr..

[79] Trull TW, Bray SG, Manganini SJ, Honjo S, François R (2001). Moored sediment trap measurements of carbon export in the Subantarctic and Polar Frontal zones of the Southern Ocean, south of Australia. J. Geophys. Res..

[80] McCave IN (1984). Size spectra and aggregation of suspended particles in the deep ocean. Deep Sea Res. Part A Oceanogr. Res. Pap..

[81] Klaas C, Archer DE (2002). Association of sinking organic matter with various types of mineral ballast in the deep sea: Implications for the rain ratio. Glob. Biogeochem. Cycles.

[82] Vázquez M (2016). Regolith production and chemical weathering of granitic rocks in central Chile. Chem. Geol..

[83] Lugmair GW, Galer SJG (1992). Age and isotopic relationships among the angrites Lewis Cliff 86010 and Angra dos Reis. Geochim. Cosmochim. Acta.

[84] Cohen AS, O’Nions RK, Siegenthaler R, Griffin WL (1988). Chronology of the pressure-temperature history recorded by a granulite terrain. Contr. Mineral. Pet..

[85] Horwitz EP, Chiarizia R, Dietz ML (1992). A novel strontium-selective extraction chromatographic resin. Solvent Extr. Ion. Exch..

[86] Pin C, Zalduegui JS (1997). Sequential separation of light rare-earth elements, thorium and uranium by miniaturized extraction chromatography: application to isotopic analyses of silicate rocks. Anal. Chim. Acta.

[87] Tanaka T (2000). JNdi-1: a neodymium isotopic reference in consistency with LaJolla neodymium. Chem. Geol..

[88] Jweda J, Bolge L, Class C, Goldstein SL (2016). High precision Sr-Nd-Hf-Pb isotopic compositions of USGS reference material BCR-2. Geostand. Geoanal. Res..

[89] Weis D (2006). High-precision isotopic characterization of USGS reference materials by TIMS and MC-ICP-MS. Geochem. Geophys. Geosyst..

[90] Jacobsen SB, Wasserburg GJ (1980). Sm-Nd isotopic evolution of chondrites. Earth Planet. Sci. Lett..

[91] Thirlwall MF (1991). Long-term reproducibility of multicollector Sr and Nd isotope ratio analysis. Chem. Geol..

[92] Albarède F (2004). Precise and accurate isotopic measurements using multiple-collector ICPMS. Geochim. Cosmochim. Acta.

[93] Galer SJG, Abouchami W (1998). Practical application of lead triple spiking for correction of instrumental mass discrimination. Mineral. Mag..

[94] Orsi AH, Whitworth III T, Nowlin WD (1995). On the meridional extent and fronts of the Antarctic Circumpolar Current. Deep Sea Res. Pt. I Oceanogr. Res. Pap..

[95] Schlitzer, R. *Ocean Data View* odv.awi.de (2019).

[96] Lang Farmer G, Licht K, Swope RJ, Andrews J (2006). Isotopic constraints on the provenance of fine-grained sediment in LGM tills from the Ross Embayment, Antarctica. Earth Planet. Sci. Lett..

[97] Hart SR (1984). A large-scale isotope anomaly in the Southern Hemisphere mantle. Nature.

