# Supplementary material for: A circumpolar dust conveyor in the glacial Southern Ocean
Source: Nat Commun. 2020 Nov 9;11:5655. doi: 10.1038/s41467-020-18858-y (PMC7652835; doi:10.1038/s41467-020-18858-y)
Supplement: Supplementary file 1 — Supplementary Information [file 41467_2020_18858_MOESM1_ESM.pdf]

Supplementary Information for

A circumpolar dust conveyor in the glacial Southern Ocean

By Struve, T., et al.

Supplementary Figure 1: Sample locations in potential dust source areas of the Southern Hemisphere

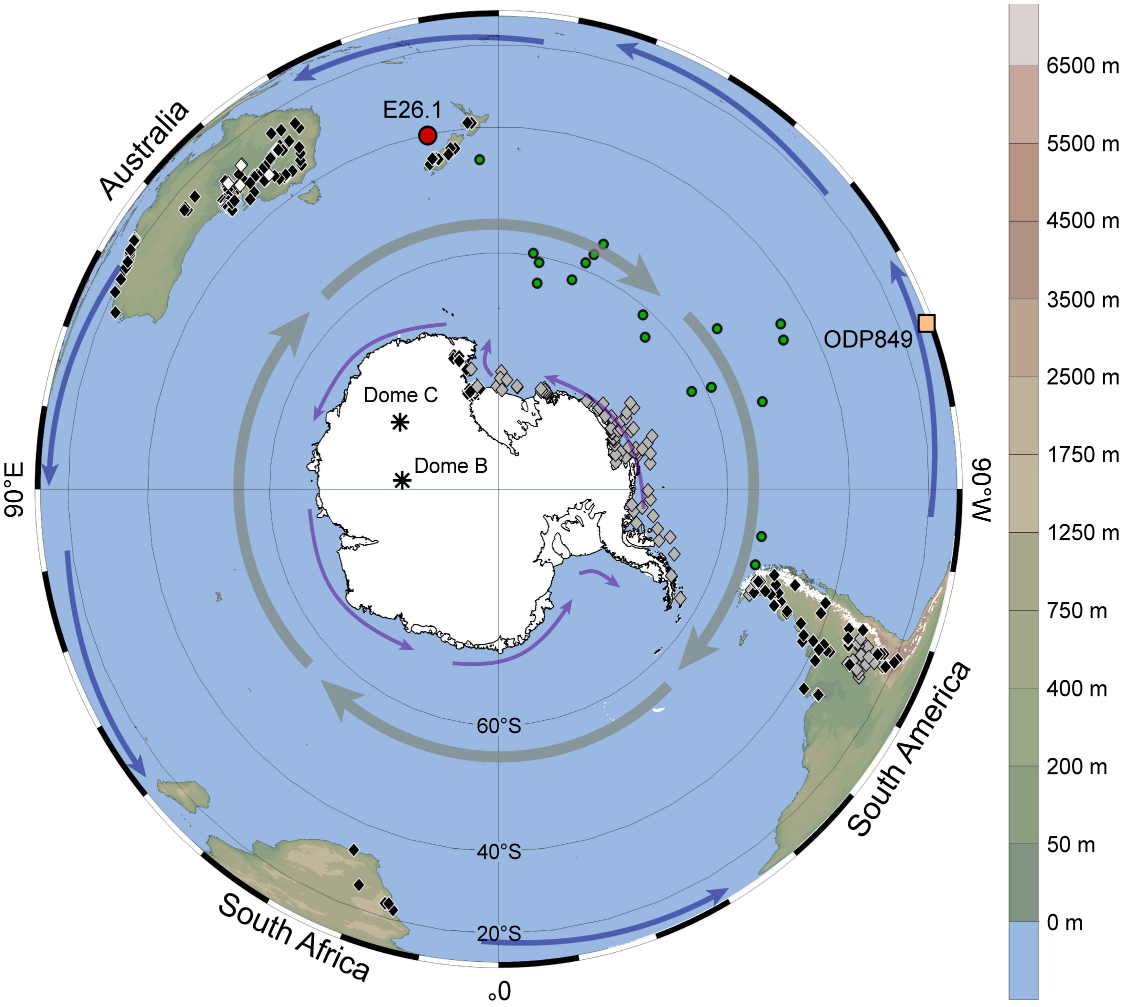

Supplementary Figure 1: Sample locations in potential dust source areas of the Southern Hemisphere. Simplified atmospheric circulation patterns indicated by dark blue (low-latitude easterly trade winds), gray (mid-latitude Southern Hemisphere westerly winds) and purple arrows (polar easterly winds around Antarctica); not including winds over the Antarctic continent. Black diamonds: dust fraction data from potential source dust areas (<2, <5, <10 µm), gray diamonds: <63 µm fraction data, white diamonds: bulk sediment samples from Australia[17], red filled circle: Eastern Tasman Sea core E26.1[52], peach filled square: ODP Leg 138, Site 849[63], green filled circles: Last Glacial Maximum sample locations (see also Fig. 1 of the main text). Large asterisks mark locations of Antarctic ice cores from Dome B and C[17,26,62]. Antarctic ice expansion produced using data from the SCAR Antarctic Digital Database. White dots indicate glacier locations (symbol size does not scale with glacier extend)[64]. Note that not all geochemical tracers are available for every sample location (see Supplementary Tables 2 and 3 for further details). Map generated using Ocean Data View[65].

Supplementary Methods

Trace element analyses were carried out on an aliquot of the fully digested sample in 2% HNO_3 matrix using a ThermoFinnigan *Element II*™ ICP-MS. To account for changes in the instruments operating conditions, all samples were doped with a beryllium-indium spike for internal normalization. Oxide formation was monitored during instrument tuning and with a synthetic

solution containing Ba, La and Ce at the beginning of the analytical session. The effect of La and Ce oxide interferences was insignificant and therefore not corrected for. However, we corrected for Ba oxide interferences on Eu isotopes due to the pronounced difference of Ba content compared to Eu (typical sample Ba/Eu of ~900). Full procedural blanks were <1 % and therefore, no blank correction was applied. Analytical precision and accuracy were determined using unleached USGS rock reference material BCR-2 (n = 10) and repeat analyses of sample C7 (PS75/059-2, 57.5–58 cm; n = 11). For all elements analyzed during this study, the 2RSD precision was better than 9 and 11 % for BCR-2 and C7, respectively (see Supplementary Table 1). The accuracy of BCR-2 rock reference material was better than 11% for all elements compared to reported reference values[1]. Analyses of HCl/EDTA-leached ground rock reference materials showed low accuracy and precision for rare earth element (REE) concentrations. In comparison to the REE reference values[1], the leached residues of BCR-2 and AGV-1 powders yielded REE depletion of up to 65% and 84%, respectively. Moreover, the depletion is characterized by a systematic fractionation along the REE series, except for Eu with concentrations of ~20–25 % below the literature values (see Supplementary Table 1).

Supplementary Table 1: Trace element results for repeat analyses of rock digests. (A) Unleached and HCl/EDTA-leached residues of USGS rock reference material BCR-2. (B) HCl/EDTA-leached residues of USGS rock reference material AGV-1. The average values of leached residues and their 2SD were calculated from repeat analyses (BCR-2: n = 23; AGV-1: n = 24) of eight individual rock digests. The unleached BCR-2 results were obtained from repeat analyses (n = 10) of a stock solution prepared from five individual BCR-2 digests. Note the systematic depletion of REE in leached rock residues compared to reference results, calculated as offset to reference values in percent. CL: confidence level (GeoReM 8689)[1]. (C) Sample C7 (PS75/059-2, 57.5–58 cm) results from n = 11 repeat analyses.

**(A) BCR-2**	**Sr**	**Zr**	**Ba**	**La**	**Ce**	**Pr**	**Nd**	**Sm**	**Eu**	**Gd**	**Tb**	**Dy**	**Ho**	**Er**	**Tm**	**Yb**	**Lu**	**Pb**
**Unleached average**	**332**	**166**	**670**	**25.8**	**51.3**	**6.7**	**28.0**	**6.43**	**1.88**	**6.39**	**1.08**	**6.54**	**1.31**	**3.71**	**0.53**	**3.33**	**0.51**	**11.6**
**2SD**	10	4	20	1.0	2.5	0.3	1.9	0.42	0.18	0.18	0.04	0.36	0.07	0.24	0.04	0.24	0.05	0.5
**2RSD**	3	2	3	4	5	4	7	7	9	3	3	5	6	7	8	7	9	5
**Leached average**	**333**	**163**	**670**	**10.2**	**19.3**	**2.4**	**10.2**	**2.56**	**1.54**	**3.00**	**0.53**	**3.50**	**0.75**	**2.31**	**0.36**	**2.38**	**0.38**	**7.9**
**2SD**	27	30	88	1.2	2.4	0.2	1.3	0.24	0.16	0.42	0.05	0.32	0.08	0.30	0.05	0.24	0.05	1.4
**2RSD**	8	18	13	12	12	9	13	9	10	14	9	9	10	13	13	10	13	18
**Reference values**	337	187	684	25.1	53.1	6.8	28.3	6.5	2.0	6.8	1.1	6.4	1.3	3.7	0.5	3.4	0.5	10.6
**95% CL (%)**	2.0	0.8	0.7	0.6	0.6	0.6	1.3	0.7	1.2	1.1	2.4	0.9	0.8	1.0	1.1	1.1	1.5	1.6
**Offset to ref. val. (%)**	-1	-13	-2	-59	-64	-65	-64	-61	-22	-56	-51	-46	-43	-37	-33	-30	-25	-25

**(B) AGV-1**	**Sr**	**Zr**	**Ba**	**La**	**Ce**	**Pr**	**Nd**	**Sm**	**Eu**	**Gd**	**Tb**	**Dy**	**Ho**	**Er**	**Tm**	**Yb**	**Lu**	**Pb**
**Leached average**	**634**	**221**	**1138**	**10.2**	**14.9**	**1.5**	**5.2**	**0.95**	**1.28**	**0.94**	**0.14**	**0.90**	**0.19**	**0.58**	**0.09**	**0.68**	**0.11**	**11.0**
**2SD**	166	25	222	1.9	2.9	0.3	1.2	0.19	0.25	0.24	0.03	0.17	0.04	0.12	0.02	0.15	0.02	2.6
**2RSD**	26	11	19	19	19	22	23	20	19	26	23	19	20	21	22	22	20	24
**Reference values**	661	232	1218	38.2	68.6	8.3	32.1	5.8	1.7	4.9	0.7	3.6	0.7	1.8	0.3	1.7	0.3	36.4
**95% CL (%)**	0.6	1.0	0.6	0.7	0.8	1.3	1.0	1.0	2.1	1.4	1.6	1.1	1.5	1.0	2.6	0.9	1.7	1.2
**Offset to ref. val. (%)**	-4	-5	-7	-73	-78	-82	-84	-84	-23	-81	-79	-75	-73	-68	-65	-59	-57	-70

**(C) Sample C7**	**Sr**	**Zr**	**Ba**	**La**	**Ce**	**Pr**	**Nd**	**Sm**	**Eu**	**Gd**	**Tb**	**Dy**	**Ho**	**Er**	**Tm**	**Yb**	**Lu**	**Pb**
**Average**	**225**	**124**	**768**	**19.0**	**37.6**	**4.4**	**17.3**	**3.4**	**0.78**	**3.10**	**0.50**	**3.07**	**0.62**	**1.85**	**0.28**	**1.84**	**0.28**	**5.4**
**2SD**	7	3	11	1.0	1.5	0.2	1.1	0.2	0.07	0.18	0.02	0.16	0.04	0.12	0.03	0.17	0.03	0.4
**2RSD**	3	3	1	5	4	5	6	5	9	6	4	5	6	6	10	9	11	7

Supplementary Note 1: Caveats and benefits of the applied strong acid leaching protocol

Our Sr, Nd and Pb isotope data show excellent reproducibility of literature values for BCR-2 and AGV-1 rock reference materials (see Methods section of the main text). However, complementary REE results from the same HCl/EDTA-leached BCR-2 and AGV-1 powders show poor accuracy and precision (see Supplementary Table 1), and a depletion relative to literature values which increases systemically from the light REE to the heavy REE (see Supplementary Table 1). The supernatant of the first 1 M HCl leaching step showed high REE content and a fractionation along the REE series which is inverse to the residual silicate fraction (Supplementary Fig. 2). This suggests that leaching with 1 M HCl removed substantial amounts of REE from the ground rock powders, in contrast to untreated digests of BCR-2 and AGV-1 powders processed in the ICBM lab yielding accurate REE concentrations[2] (see also Supplementary Methods).

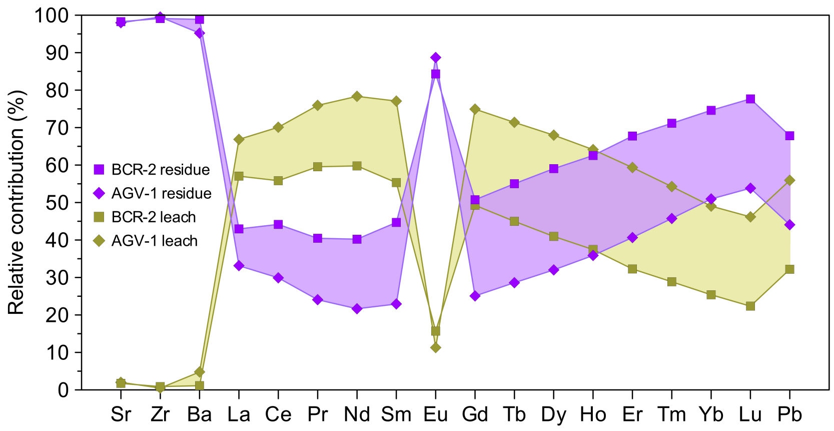

Supplementary Figure 2: Trace element content of leach and residual fractions of BCR-2 and AGV-1 powders after HCl/EDTA leaching. BCR-2 and AGV-1 residue values are averages of n = 3 repeat analyses, respectively. Element concentrations are presented in percent relative to the sum of the two fractions. Leach fractions were taken after ~3 h exposure to 1 M HCl at room temperature (see also Methods section of the main text).

Interestingly, the divalent cations Sr, Zr, Ba and Eu are significantly less affected (Supplementary Fig. 2) and their concentrations are close to published literature values (Supplementary Table 1). The good agreement of Zr concentrations in acid-leached rock powders with reference values suggests that our digestion protocol was successful in decomposing the highly resistant zircon mineral structures. Some of the observed differences in trace metal concentrations between HCl-leached and unleached BCR-2 and AGV-1 powders (Supplementary Fig. 2) may be ascribed to the removal of contaminants introduced during rock crushing and pulverization[3]. However, similar REE concentrations in first and second generation BCR and AGV powders indicate that the effect of contamination is small for the REE[3,4]. Instead, we suggest that degradation of REE-bearing mineral structures during rock pulverization favored the increased mobilization of REE from the solid phase into solution during HCl leaching.

Remarkably, REE results of HCl/EDTA-leached South Pacific surface samples do not show evidence for systematic fractionation along the REE series in comparison to HH/AcOH leaching[2], an approach typically applied to minimize attacking silicate mineral structures[5]. Consequently, we follow the arguments of ref. 2 and suggest that REE in intact silicate mineral structures of the <5 µm sediment fraction are largely unaffected by our HCl/EDTA-based leaching protocol. This is consistent with earlier work showing that most clay minerals (<2 µm size fraction) remain largely intact after exposure to 6.45 M HCl over 10 days[6], i.e. a significantly longer reaction time and higher acid concentration than in our study. Likewise, HCl leaching shows little to no effect on important rock forming silicate minerals[7], in that their etching/dissolution usually requires hydrofluoric acid[7,8]. Importantly, HCl/EDTA was found to be more efficient than HH/AcOH in removing authigenic precipitates and/or laboratory contaminants from the silicate fraction of marine sediments processed for dust provenance studies[2]. Reliable extraction of a lithogenic signal is particularly important in depositional environments where the lithogenic component is strongly diluted by authigenic precipitates, such as in South Pacific calcareous and siliceous microfossil oozes[2,9,10,11,12,13]. In these environments, incomplete removal of trace metal rich authigenic precipitates can represent a significant fraction of the total sedimentary trace metal budget thus obscuring a low abundance terrestrial input signal.

The flat patterns of our shale-normalized REE data are clearly distinct from quantitatively important authigenic REE carrier phases (ferromanganese (oxy)hydroxides, marine barites and calcium carbonate) and hydrothermal output (Supplementary Fig. 3a). This demonstrates that the applied leaching procedure extracted the lithogenic signal from our South Pacific marine sediment samples. Furthermore, our HCl/EDTA-leached BCR-2 and AGV-1 rock digests reproduce previous strong-acid leaching approaches to remove the Pb contamination introduced during rock crushing and pulverization[3,14], whereas HH/AcOH leaching was shown to be insufficient to fully remove this Pb contamination[2]. This would consequently compromise the use of BCR-2 and AGV-1 reference materials for external quality control during our Pb isotope analyses.

We therefore conclude that HCl/EDTA leaching (i) does not remove REE and other trace metals from intact silicate mineral structures, (ii) is efficient in extracting the (lithogenic) silicate fraction from South Pacific marine sediments, and (iii) improves the assessment of external reproducibility in particular for Pb isotope analyses[2,14].

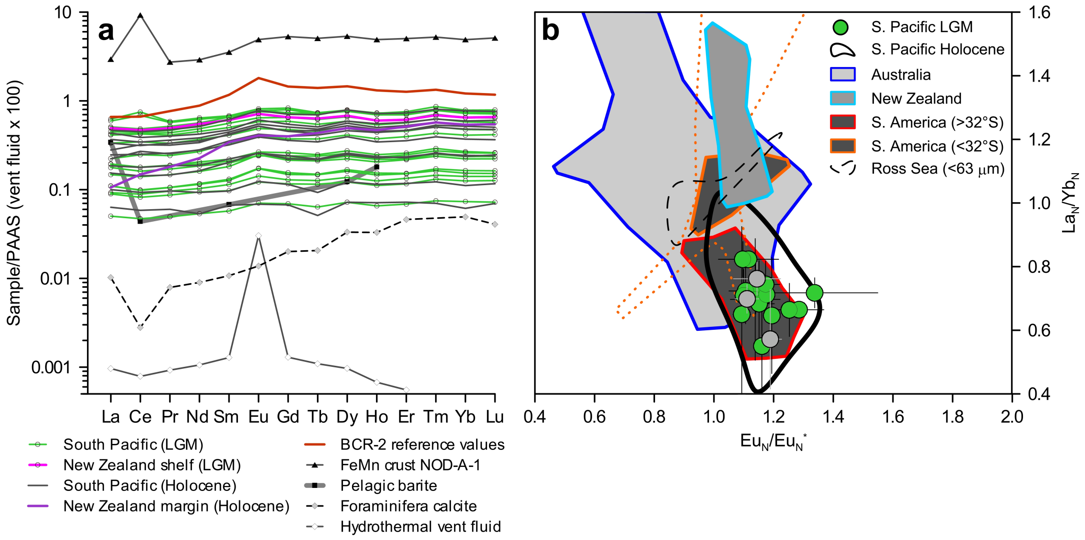

Supplementary Figure 3: Rare earth element results of South Pacific fine fraction samples. a South Pacific fine fraction rare earth element (REE) data normalized to Post-Archean Australian Shale (PAAS)[16] shown as averages per location for the Last Glacial Maximum (LGM) (see Supplementary Data File 2) and single sample results for the Holocene (Supplementary Data File 3). The results are compared with USGS rock reference material BCR-2[1], hydrothermal vent fluids[66] and important authigenic phases in marine sediments including hydrogenetic ferromanganese precipitates (USGS reference material NOD-A-1)[67], pelagic barite (physically separated microcrystals, sample Proa 101)[68] and foraminifera calcite (cleaned coretop *G. sacculifer* from Southeast Pacific core 54MC)[69]. **b** South Pacific LGM fine fraction REE results illustrated as shale-normalized (subscript `N´) La/Yb ratio versus europium anomaly ($Eu/Eu_N^* = 3 \times Eu_N/[2 \times Sm_N + Gd_N]$). LGM data points and error bars represent averages and their 2SD across the LGM interval (~18–24 ka BP). Green: open ocean locations; gray: locations off New Zealand and South America. Literature potential source area (PSA) data from South America (<5 µm)[15], New Zealand (bulk loess, glacial dust, dust traps)[58], East Australia (river sediments of <25 µm, <80 µm, <90 µm size fractions, and bulk sediment)[42,53,58,70], and the Holocene mid-latitude South Pacific (<10 µm)[2]. Note that the published REE data may not represent the full range of Southern Hemisphere PSA compositions. For example, South America (<32°S) comprises six <5 µm fraction samples plotting in a confined space in **b**, whereas the <63 µm fraction from the same region comprises 19 samples[15] which occupy a significantly larger space (stippled orange polygon).

Supplementary Note 2: Rare earth elements as provenance tracers in the study area

The REE content of our South Pacific <5 µm lithogenic sediment samples provides independent provenance information (Supplementary Fig. 3b). The merit of analyzing REE is a discrimination of published New Zealand PSA REE data. Despite known grain size related fractionation effects along the REE series[15] and the large range of size fractions investigated, published terrestrial PSA REE data from New Zealand are relatively homogeneous as reflected in their confined extent in La/Yb_N – Eu/Eu_N^* space (normalized to PAAS)[16] (Supplementary Fig. 3b). As such, available New Zealand data are clearly distinct from our <5 µm fraction REE compositions (Supplementary Fig. 3b), including samples from core SO213/082-1 located east of New Zealand (see Supplementary Note 3 for more details). This distinction indicates the potential of using REE as a dust provenance tracer in the study area. However, a comprehensive REE-based dust provenance investigation requires better characterization of REE geochemistry in Southern Hemisphere dust PSAs.

Supplementary Note 3: Sample locations near the continents

Our sample set contains three sampling locations which were selected to characterize LGM input signals from nearby New Zealand and Southern South American PSAs (main text Fig. 1). In particular, the <5 μm fraction from core SO213/082-1 retrieved in the Bounty Trough east of New Zealand (2066 m water depth) yielded highly radiogenic Pb isotope compositions ($^{206}Pb/^{204}Pb$ of 19.306 ± 0.085, 2SD, n = 5; Supplementary Fig. 4). These compositions are lower than nearby shelf (669 m water depth) $^{206}Pb/^{204}Pb$ of 19.445 ± 0.002 from site PS75/105-1[2] (Supplementary Figs. 4 and 5), but inconsistent with terrestrial Pb isotope data from New Zealand's South Island[17]. A similar difference between the New Zealand sources and the material deposited at SO213/082-1 is evident from our REE data (Supplementary Fig. 3b). In contrast, both coretop[2] and LGM Nd and Sr isotope compositions (main text) are in agreement with terrestrial source signatures in New Zealand.

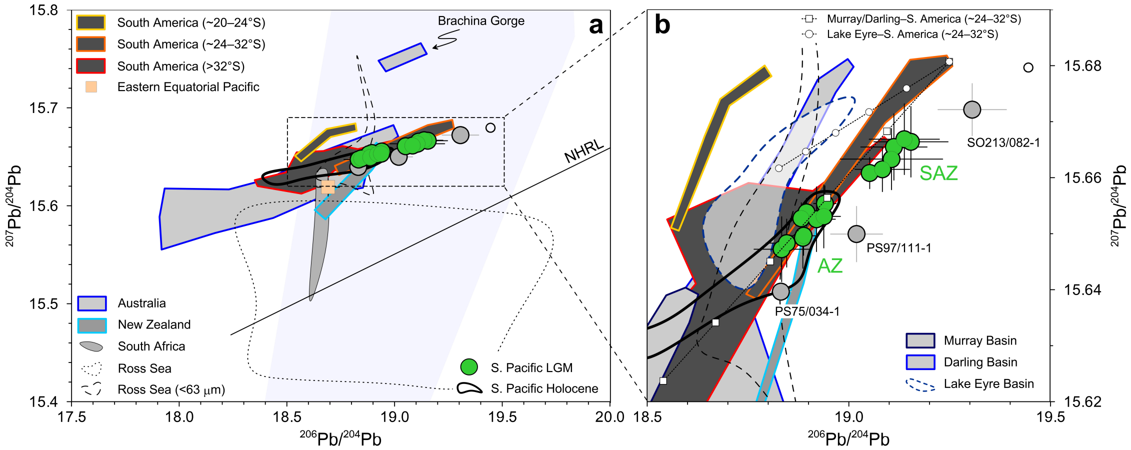

Supplementary Figure 4: South Pacific Last Glacial Maximum fine fraction data in $^{207}Pb/^{204}Pb$-$^{206}Pb/^{204}Pb$ space. Last Glacial Maximum (LGM) sample symbols represent average values of typically five individual data points and the error bars of their respective 2SD across the LGM interval (18–24 ka BP). Remote open ocean samples in green, locations at/near the continental margins in gray. Black ring indicates coretop sample PS75/105-1 from east of New Zealand[2]. **a** Overview of Southern Hemisphere potential source area (PSA) dust fraction compositions. Average LGM value for the Eastern Equatorial Pacific from ref. 63. Light blue shading indicates Dome C ice core data[17]. See Supplementary Table 3 for a full list of references to PSA data. NHRL: Northern Hemisphere Reference Line[71]. **b** Detailed view including endmember mixing models. Filled white symbols represent 20 % increments of mixing of Central South America (24–32°S) with Lake Eyre (circles) and Murray-Darling basin endmembers (squares), respectively. See Supplementary Table 2 for endmember details. Note the poor alignment of Lake Eyre mixing with our South Pacific data in comparison to $^{208}Pb/^{204}Pb$–$^{206}Pb/^{204}Pb$ space (Fig. 3 of the main text). Lake Eyre PSA samples have been revisited with results showing similar $^{206}Pb/^{204}Pb$, but significantly lower $^{207}Pb/^{204}Pb$ which are consistent with our mixing scenarios[72].

In the absence of local terrestrial PSA data which could provide an explanation for these highly radiogenic Pb isotope compositions east of New Zealand, we ascribe these results to mineral sorting effects during riverine, coastal and/or submarine mass transports[18] which are abundant in the Bounty Trough[19]. Sorting effects have also been suspected to influence the Sr isotope compositions of bulk sediments east of New Zealand, highlighting that these processes need to be

considered for the interpretation of provenance data[12]. Hydrodynamic/gravitational sorting can lead to enrichment of heavy minerals with highly radiogenic Pb isotope compositions, which can induce substantial Pb isotope changes in sediments with minor to moderate effects on Nd and Sr isotope compositions[18]. Mobilization and transport of these sediments (airborne and/or submarine) could carry a radiogenic Pb isotope signal offshore into the South Pacific SAZ. Submarine transport of sediments from the Bounty Trough along the entire South Pacific SAZ appears unlikely, because the downslope sediment transport exiting the Bounty Trough is carried northwards from the Bounty Fan area by the Deep Western Boundary Current[19,20]. If the radiogenic Pb isotope signal was also present in shallow shelf sediments, the lowered LGM sea-level could have exposed these sediments to deflation. However, the offshore transport of the New Zealand shelf/margin signal in seawater suspension and/or airborne should be reflected in a zonal Pb isotope gradient in the study area as a result of preferential settling of heavy minerals during transport[18]. This is not evident from the available data so that the radiogenic Pb isotope signal east of New Zealand is considered a local feature.

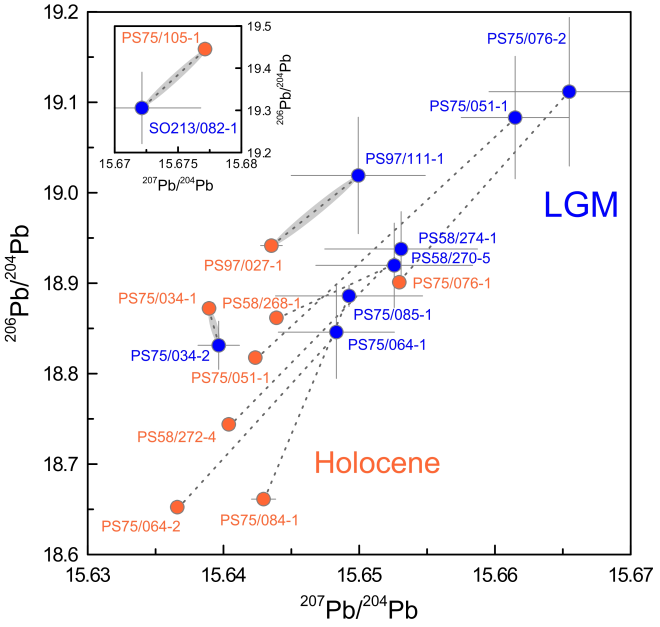

Supplementary Figure 5: Comparison of Last Glacial Maximum and Holocene Pb isotope data pairs. Stippled lines connect Holocene[2] (orange) and Last Glacial Maximum (LGM) (blue) (this study) data pairs from the same sampling locations. Where LGM and Holocene data could not be paired at the exact same locations, nearby locations are used for comparison. Locations at or near the continental margins of South America and New Zealand (inset in top left corner) are illustrated with gray shading. Holocene Pb isotope data from PS75/084-1 obtained from the <5 µm fraction (see Supplementary Note 5). Note that the LGM-to-Holocene shift is moderately inverse at location PS75/097 (not shown here, see Supplementary Data Files 2 and 3). LGM data points and error bars represent averages and their 2SD across the LGM interval (~18–24 ka BP). Holocene data points are single measurements and the error bars represent the 2SD of repeated analyses of secondary rock reference materials as reported by ref. 2. No error bars are shown where the 2SD is smaller than the symbol size.

At site PS97/111, the difference between terrestrial PSAs in Southern Patagonia and ocean margin sediment Pb isotope compositions seems relatively small (see Supplementary Fig. 4) compared to the New Zealand shelf. Yet, the geochemistry of fine material input from the western flank of the South Patagonian Andes is largely unconstrained. Therefore, we can only speculate if local input and riverine/coastal hydrodynamic mineral sorting played a role on the South American shelf in analogy to the sediments deposited east of New Zealand. Interestingly, the Nd, Sr and Pb isotope characteristics of <5 μm fraction samples from PS75/034 further offshore are also consistently different to the remaining dataset (Supplementary Data Files 1−3). The nearly invariable LGM–Holocene Pb isotope compositions at site PS75/034 (Supplementary Fig. 5) are similar to available Patagonian PSA data (Supplementary Fig. 4). A contribution from volcanic sources in Patagonia is also consistent with more radiogenic ε_{Nd} of -3.7 ± 0.5 and less radiogenic $^{87}Sr/^{86}Sr$ of 0.7083 ± 0.0005 at PS75/034 (2SD, n = 5; Fig. 2 and Supplementary Data File 2). This suggests that the signal at site PS75/034 is probably influenced by mineral dust emitted from terrestrial sources in Patagonia. The transport of dust is presumably related to wind anomalies reaching several hundred kilometers into the Southeast Pacific against the predominating westerly winds[21].

Supplementary Table 2: Average values for main potential dust source areas in the Southern Hemisphere. Values were calculated using results of <5 μm fraction sediment material where available. See original publications and Supplementary Table 3 for details about grain size fractions of the radiogenic isotope data. The Nd and Sr isotope data of two samples from Tasman Sea core E26.1 (40.28°S, 168.33°E)[52] were combined with central Australian Pb isotope data[17] to constrain an integrated dust signal exported from Australia. Australian clay fraction Pb isotope data with elevated Pb/metal ratios are excluded[40]. Source data in bold are included in isotope mixing calculations illustrated in Figs. 3, 4 and Supplementary Fig. 4. Isotope and concentration data from the same fine fraction sample material were considered preferentially. Where combined datasets are unavailable, Pb, Nd and Sr concentration data were derived from additional samples and/or grain size fractions (see original publications for further details).

	$^{206}Pb/^{204}Pb$	2SD[a]	$^{207}Pb/^{204}Pb$	2SD	$^{208}Pb/^{204}Pb$	2SD	n[b]	[Pb] (μg/g)	ε_{Nd}	2SD	n	[Nd] (μg/g)	$^{87}Sr/^{86}Sr$	2SD	n	[Sr] (μg/g)	References
**Lake Eyre Basin[*]**	**18.83**	**0.27**	**15.66**	**0.02**	**38.74**	**0.30**	**9**	**11.3**	**-4.1**	**2.0**	**17**	**23.3**	**0.7112**	**0.0038**	**17**	**166**	17,52−54
**Murray Basin**	**18.48**	**0.20**	**15.62**	**0.03**	**38.50**	**0.20**	**8**	**17.9**	**-9.1**	**3.4**	**24**	**36.6**	**0.7316**	**0.0331**	**24**	**90**	42,54−56
**Darling Basin**	**18.60**	**0.61**	**15.62**	**0.07**	**38.60**	**0.69**	**11**	**19.3**	**-2.6**	**4.2**	**19**	**28.2**	**0.7108**	**0.0028**	**19**	**109**	17,42,54−56
South Australia[‡]	–	–	–	–	–	–	–	22.4	-12.2	6.1	38	24.5	0.7200	0.0119	38	225	42,52−54
West Australia	17.95	0.06	15.61	0.02	38.02	0.26	3	–	-17.0	8.3	21	22.5	0.7381	0.0450	21	190	52,54,55,57
**Tasman Sea (core E26.1)**	**18.85**	**0.25**	**15.66**	**0.02**	**38.80**	**0.35**	**12**	**15.3**	**-5.1**	**1.4**	**2**	**25.7**	**0.7115**	**0.0012**	**2**	**157**	17,42,52−56
New Zealand, South Island	18.82	0.26	15.63	0.06	38.72	0.35	3	16.2	-5.4	3.7	10	27.2	0.7142	0.0103	10	293	17,26,58
S. America 20-24°S[+]	18.69	0.23	15.67	0.03	38.85	0.15	3	16.7	-10.5	1.8	3	13.5	0.7150	0.0079	3	372	15,30
**S. America 24-32°S (selected)[#]**	**19.25**	**0.002**	**15.68**	**0.002**	**39.31**	**0.006**	**1**	**16.7**	**-5.0**	**0.2**	**2**	**20.9**	**0.7169**	**0.00002**	**1**	**172**	15,30,38
S. America 24-32°S (total)[§]	*(18.96)*	*(0.40)*	*(15.66)*	*(0.04)*	*(38.90)*	*(0.44)*	*(6)*	*(16.7)*	*(-4.7)*	*(1.9)*	*(6)*	*(18.6)*	*(0.7160)*	*(0.0126)*	*(6)*	*(172)*	15,30,38
S. America >32°S	18.70	0.30	15.64	0.03	38.65	0.33	21	15.2	-1.3	6.7	27	14.5	0.7102	0.0148	27	109	15,30,38,59,60
South Africa	18.65	0.05	15.59	0.12	38.33	0.22	3	–	-12.5	11.9	6	–	0.7260	0.0209	6	–	17,26
Ross Sea dust	18.72	0.98	15.53	0.15	38.86	1.51	8	13.0	-1.8	10.1	43	36.7	0.7109	0.0174	46	254	17,26,36[†],37
Ross Sea shelf (<63 μm)	18.76	0.19	15.65	0.08	38.79	0.30	9	15.1	-8.0	4.4	18	34.3	0.7202	0.0085	18	147	36
West Antarctic shelf (<63 μm)	–	–	–	–	–	–	–	23.0	-4.1	7.1	66	32.6	0.7156	0.0259	54	200	61

[*] Brachina Gorge full digest samples contain a soluble fraction with highly radiogenic Pb isotope compositions and are therefore not included in calculating the endmember values[17]. Not including Coober Pedy Nd and Sr isotope data[52].

[+] [Sr] adopted from S. America >32°S[15].

[#] Pb isotope endmember values defined based on <63 μm N-CWA sample 8 in ref. 30. Nd isotope data averaged from <63 μm and <5 μm results for sample 13 in ref. 15, i.e. sample 8 in ref. 30. Sr isotope data included only from <5 μm fraction to reduce bias from grain size effect. [Sr] adopted from S. America >32°S[15].

[§] Calculated using available <5 μm fraction data between ~24 and 32°S and including <63 μm for N-CWA Pb isotope compositions from regions where no <5 μm fraction data are available[15,30]. [Sr] adopted from S. America >32°S[15].

[†] Concentration data adopted from terrestrial <63 μm fraction data[36].

[a] 2 standard deviations (2SD) of respective PSA sample population.

[b] Number of samples used to constrain source area characteristics (see also Supplementary Fig. 1).

Supplementary Table 3: Grain size details for radiogenic isotope data from Southern Hemisphere potential source areas used in Figs. 2, 3, 4 and Supplementary Fig. 3. See also Supplementary Fig. 1 for the distribution of samples from potential dust source areas in the Southern Hemisphere.

	Grain size fraction	Nd-Sr isotope data references	Pb isotope data references
**South America**	<5 µm	15,60	17§,30,59
**South Africa**	<5 µm	26	17
**Australia**	<5 µm	52	–
**Australia**	bulk#	–	17
**Australian clay fraction**	<2 µm	54,55	54,56
**New Zealand**	<5 µm	26	17
**Ross Sea (dust)**	<5 µm	26,37	17
**Ross Sea (shelf)**	<63 µm	36	36
**West Antarctica**	<63 µm	61	–
**South Pacific coretop sediments**	<10 µm	2	2
**EPICA Dome B and C**	<5 µm	26,62	17
**Eastern Equatorial Pacific**	bulk	–	63

§South American data from ref. 17 used only in Fig. 5 (including two bulk sample results).
#No physical grain size separation applied for samples analyzed for their Pb isotope composition. Results of bulk HF-digested samples used[17].

Supplementary Table 4: Summary of the tracer evidence for the provenance of dust fraction sediments in the South Pacific during the Last Glacial Maximum. Tracer evidence with caveats (in italics and brackets) related to possible bias of original provenance information by grain size effects *(Sr)* (see Supplementary Notes 4 and 5) or limited data coverage in the potential source areas (PSAs) *(REE)* (Supplementary Note 2). See Fig. 1 and Supplementary Fig. 1 for geographical locations and sample coverage of the individual PSAs. The geochemical properties of the individual PSAs are listed in Supplementary Table 2.

Potential source area	Present in AZ dust fraction	Present in SAZ dust fraction	Tracer evidence supporting PSA inclusion	Tracer evidence supporting PSA exclusion	Confidence in presence/ absence
Lake Eyre Basin, Australia	**Major component**	**Minor component**	Nd, Pb, *(Sr)*	-	High
Darling Basin, Australia	**Part of major component**	**Part of minor component**	Pb, *(Sr)*	Nd (with exceptions)#	High
Murray Basin, Australia	**No**	**No**	Pb	Nd, *(Sr)*	High
South Australia	**No**	**No**	-	Nd, Pb, *(Sr)*	High
West Australia	**No**	**No**	-	Nd, Pb, *(Sr)*	High
New Zealand, South Island	**No**	**No**	Nd, Pb, *(Sr)*	*(REE)*	Low
South America 20-24°S	**No**	**No**	-	Nd, Pb, *(Sr)*	High
South America 24-32°S	**Minor component**	**Major component**	Nd, Pb	*(Sr)*	High
South America >32°S	**No**	**No**	-	Nd, Pb, *(Sr)*	High
South Africa	**No**	**No**	-	Nd, Pb, *(Sr)*	High
Ross Sea dust	**No**	**No**	-	Nd, Pb, *(Sr)* & low dust fluxes	High
West Antarctic and Ross Sea shelves	**Minor component**	**No**	Nd, Pb, Sr*	-	High

*See Supplementary Notes 4 and 5.
#Exclusion not applicable to the entire Darling Basin (see main text for details).

Supplementary Note 4: Grain size effects on the fine fraction strontium, neodymium and lead isotope signals

The Sr isotope composition of clay minerals is primarily related to the mineral composition, age and Rb-Sr content of the parent rock/soil material[22]. Yet, sorting processes and the formation of clay minerals from weathering fluids can lead to an enrichment of radiogenic ^{87}Sr in the sedimentary fine fraction[22,23,24]. This is reflected in elevated ^{87}Sr/^{86}Sr of the sedimentary fine fraction relative to the larger grain size fractions and typically referred to as grain size effect[15,25,26,27,28,29]. Therefore, the Sr isotope composition of sediments can be influenced by changes in the weathering regime at the sediment source and/or changes in the relative abundance

of clay minerals between the source and the site of deposition. Although we minimized the effect of grain size variations by selecting the <5 μm fraction for our study, it is pointed out in the main text that the Sr isotope composition of our samples from the South Pacific SAZ may not reflect the original provenance signal. Therefore, we base critical conclusions on dust provenance primarily on Nd and Pb isotope evidence, i.e. two tracers of dust provenance which are considered less sensitive to grain size variability[15,27,30].

The effects of grain size and/or mineral sorting on the Nd isotope composition of sediments are usually modest[15,18,27,28]. However, the sedimentary Pb isotope budget can be influenced by the abundance of heavy minerals so that mineral sorting in analogy to variations in fluvial sediments[18] (see also Supplementary Note 3) could play a role for Pb isotope based provenance studies of the dust cycle. Heavy minerals are relatively enriched in the sedimentary coarse fraction[18] so that a significant heavy mineral effect should be reflected in pronounced and systematic Pb isotope offsets between the coarse and the fine fractions of sediment samples. South America is the only main dust source region in the mid-latitude Southern Hemisphere where published Pb isotope data allow a systematic comparison between sedimentary fine and coarse fractions[30,31]. The $^{206}Pb/^{204}Pb$ offset is typically less than 0.12 between the <5 and <63 μm fractions of sediments from South American PSAs (based on n = 19 samples, not including two Patagonian samples with unusually high offsets)[30,31]. The Pb isotope offsets between the two size fractions are erratic and relatively small in comparison to the total range of Pb isotope compositions in South American PSA sediments ($^{206}Pb/^{204}Pb$ from 18.36 to 19.25)[30] so that Pb isotopes are considered relatively insensitive to mineral sorting processes in the Southern Hemisphere dust cycle.

Consequently, the combination of Pb and Sr isotopes can help to differentiate between the influence of changes in dust provenance and processes controlling the grain size of dust. The individual results for our <5 μm sediment samples show relatively large scatter in Pb-Sr isotope space (Supplementary Fig. 6a). Yet, the location average values for the LGM time slice reveal systematic patterns in this scatter, such that sites with lower $^{206}Pb/^{204}Pb$ tend to be associated with higher $^{87}Sr/^{86}Sr$ (Supplementary Fig. 6b). The locations in the SAZ and Polar Frontal Zones (PFZ) show higher Pb isotope variability (i.e., higher 2SD of their $^{206}Pb/^{204}Pb$ results) compared to locations further south in the Antarctic Zone (AZ), where the $^{87}Sr/^{86}Sr$ shows higher 2SD (Supplementary Fig. 6b). The Pb isotope variability can be ascribed primarily to the dynamic changes in dust provenance discussed in the main text. Accordingly, the relatively large variability of Sr isotope compositions in the AZ could be interpreted as mixing of two sources with different Sr, but similar Pb isotope compositions (Supplementary Fig. 6a), but the Nd isotope compositions of possible Ross Sea and/or Australian PSAs are inconsistent with this idea (see main text and Supplementary Note 5 below). Therefore, we suggest that the Sr isotope variability in our LGM

South Pacific dataset reflects both changes of the dust grain size composition (i.e. primarily clay mineral abundance) and changes in particle provenance. This is consistent with earlier work reporting a high sensitivity of dust Sr isotope compositions to wind sorting[28] and suggests that the grain size composition of dust delivered to our sampling locations from more proximal sources in Australia was subject to higher variability (AZ in Supplementary Fig. 6b) than far-travelled dust from Central South America during the LGM (SAZ in Supplementary Fig. 6b).

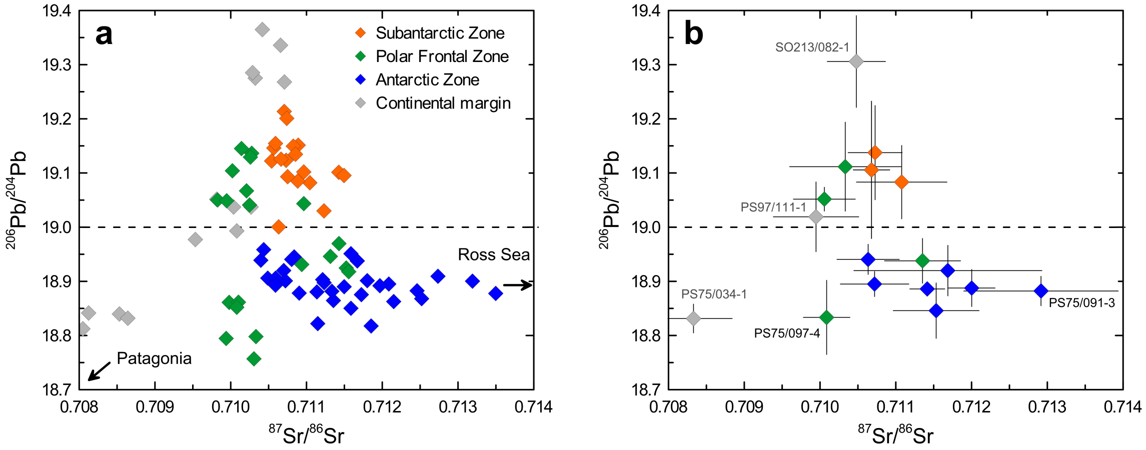

Supplementary Figure 6: South Pacific fine fraction data for the Last Glacial Maximum in Pb−Sr isotope space. a Single sample results. The dashed line demarks the range of Australian dust source Pb-Sr isotope composition ($^{206}Pb/^{204}Pb$ <19)[17]. Ross Sea and Patagonian endmember values (black arrows) would plot outside the range of the x-axis (see Supplementary Table 3 for references). **b** Average values of individual sample results shown in **a** calculated per location across the Last Glacial Maximum interval (18–24 ka BP) and plotted with their 2SD (gray bars). Symbol color coding as in **a**. Note the increased Sr isotope 2SD relative to the Pb isotope 2SD at locations in the Antarctic Zone.

Supplementary Note 5: Complementary constraints on the influence of ice-rafted detritus in the Southwest Pacific

Ice-rafted detritus (IRD) reaches the South Pacific AZ mostly from the Ross Sea[32,33]. The Ross Sea IRD can contain an originally eolian component, which is quantitatively important close to the local terrestrial sources (e.g., in the McMurdo Sound area, SW Ross Sea), but the eolian contribution to the particle fluxes decreases exponentially offshore[34]. Although it appears unlikely that IRD from the Ross Sea would be the second dominating endmember delivering lithogenic <5 µm material across the entire study area, it could represent an important source in particular for the Southwest Pacific[32,33,35]. Previous work ascribed $^{87}Sr/^{86}Sr$ of up to 0.7151 in the <63 µm fraction of glacial sediments from the Southwest Pacific to the influence of terrigenous material from the Ross Sea area[32] (Supplementary Fig. 7).

The Ross Sea endmember can be approximated by the composition of the lithogenic <63 µm fraction of glaciomarine deposits on the Ross Sea shelf[36] showing average ε_{Nd} of ~-8 and $^{87}Sr/^{86}Sr$ of ~0.7202 (Supplementary Fig. 7). Most of our glacial <5 µm fraction Sr isotope compositions are less radiogenic than both, the Ross Sea endmember[36] and Southwest Pacific[32] <63 µm

sediment fraction $^{87}Sr/^{86}Sr$ (Supplementary Fig. 7), which is at odds with the grain size effect on sedimentary Sr isotope compositions which would suggest more radiogenic Sr isotope compositions for the smaller grain size fraction[15,28,29,34,37]. Moreover, pronounced $^{87}Sr/^{86}Sr$ shifts of up to ~0.0044 are observed in the <63 µm fraction of LGM sediments along a meridional transect in the Southwest Pacific[32] (Supplementary Fig. 7), whereas Sr isotope data of our <5 µm lithogenic sediment fraction show at most moderate and gradual changes along similar meridional S-N transects during the LGM (Supplementary Fig. 7). This suggests that the two size fractions are controlled by different sediment sources and transport processes, such that the <5 µm fraction is largely unaffected by IRD deposition even near the main export route of rafting ice from the Ross Sea[33,35] (Supplementary Fig. 7). Only the Sr isotope composition of the southernmost location (core PS75/091-3) is consistent with a small contribution of <5 µm fraction material from the Ross Sea (Supplementary Fig. 7).

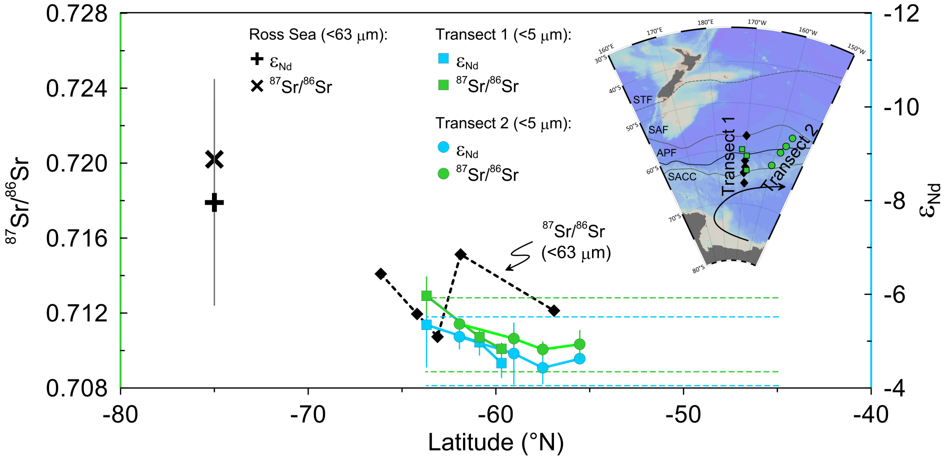

Supplementary Figure 7: The influence of Ross Sea lithogenic material on the fine fraction signal in the Southwest Pacific. Broken lines indicate the 2SD of the Sr (green) and Nd (blue) isotope compositions of our Last Glacial Maximum (LGM) open ocean samples (n = 70), respectively. Two meridional transects of <5 µm fraction data from the Southwest Pacific are depicted for comparison with existing <63 µm lithogenic sediment fraction Sr and Nd isotope data[32] (black diamonds, see inset map). Transect 1 and 2 data points and error bars represent averages and their 2SD across the LGM interval (~18–24 ka BP), uncertainty of Sr isotope data of ref. 32 smaller than symbol size. Note the unradiogenic Sr isotope composition and low latitudinal variability of the <5 µm fraction samples relative to the <63 µm transect data[32]. The Ross Sea endmember constraints (black crosses) are derived from the lithogenic <63 µm fraction of sediments from the Ross Sea shelf[36] (1SD, n = 18). The black arrow on the inset map illustrates the main ice export route from the Ross Sea[33,35].

Supplementary Note 6: Comparison of different grain size fractions in Holocene and LGM sediments

Previous dust provenance work on Holocene sediments from the mid-latitude South Pacific used the lithogenic <10 µm fraction[2]. The authors based their decision for this size fraction on the assumption that the extremely low interglacial Southern Hemisphere dust fluxes could not provide sufficient lithogenic material for dust provenance studies in the <5 µm fraction[2]. Nevertheless, sufficient material was available from some surface samples showing that Nd and Sr isotope

differences are small between the <10 µm and <5 µm fractions[2] (Supplementary Fig. 8). Here we extend the existing dataset of ref. 2 by Pb isotope analyses of the <5 µm fraction of their grain size comparison surface samples. The new surface sediment data are complemented by REE, Nd, Sr and Pb isotope analyses of <10 µm and <5 µm fractions extracted from the LGM section of core PS75/097-4 (see Supplementary Data Files 1 and 3).

The results for the <10 µm and <5 µm size fractions of PS75/097-4 LGM sediments are consistent (Supplementary Fig. 8, Supplementary Data Files 1 and 3) with earlier work that grain size induced differences are small between these two fractions in sediment samples from the mid-latitude South Pacific[2]. However, the complementary Pb isotope data of the surface samples reveal unexpected offsets between the two size fractions. The maximum offset is observed in PS75/084-1 where $^{206}Pb/^{204}Pb$ is 0.26 higher in the <5 µm fraction than in the <10 µm fraction (Supplementary Fig. 8). Conversely, $^{206}Pb/^{204}Pb$ is lower by up to 0.17 in the coretop <5 µm fraction of PS75/064-2, PS58/272-4, PS2699-5 and PS75/034-1 (Supplementary Fig. 8). The inverse offset in PS75/084-1 could be related to a different mineralogy and/or to the low lithogenic content[2,11] explaining also the remarkably low Pb yield at this location (~2 ng Pb out of 51.1 mg <10 µm fraction sample material). Despite this low yield, a worst-case scenario blank correction using the Pb isotope composition of our procedural blank would change the $^{206}Pb/^{204}Pb$ only by ~0.03 (3.4% blank contribution with $^{206}Pb/^{204}Pb$ = 17.72), thus not accounting for the observed offset of 0.26. It therefore remains unclear whether the PS75/084-1 <10 µm fraction sample was affected by Pb contamination we could not monitor and/or sample heterogeneity. Considering the extremely low abundance of lithogenic fine fraction material in this coretop sample, it seems likely that sample heterogeneity played a role. We therefore focus on the open ocean samples available for comparison of the <5 µm and <10 µm grain size fractions.

Notwithstanding the limited number of open ocean surface samples (n = 4) available for a comparison of the <5 µm and <10 µm grain size fractions, we observe a systematic offset towards less radiogenic Pb isotope compositions in the <5 µm fraction (Supplementary Fig. 8). This offset is not reproduced by the complementary Nd and Sr isotope results (Supplementary Fig. 8). The sample locations are distributed across the entire mid-latitude South Pacific and from water depths between ~4500 and 5100 m. Due to the different characteristics between the individual sampling locations, we consider hydrodynamic or gravitational mineral sorting processes in analogy to the New Zealand shelf Pb isotope signal unlikely (see Supplementary Note 3). Owing to the low amount of available surface sample material, sample weights of the <5 µm fraction surface samples were only between 16.0 and 34.8 mg, whereas the <10 µm fraction weights conform to our target of ~50 mg (Supplementary Data File 3). The Pb concentrations are higher in the <5 µm sediment fractions than in the <10 µm fractions, presumably related to the reduced abundance of

opal debris in the <5 μm fraction samples. We note slightly elevated Pb/La in the Holocene <5 μm fraction samples (Pb/La of 0.62, n = 4, not including shelf sample PS97/027-1) compared to the <10 μm fraction (Pb/La of 0.47, n = 11, not including shelf location PS75/105-1) and LGM <5 μm fraction samples (Pb/La of 0.44, n = 73, not including shelf/margin samples from SO213/082-1 and PS97/111-1). Yet, these ratios are similar to the average upper continental crust values[16] of 0.67 and in agreement with Pb/La of uncontaminated PSA soil samples from South America[38,39] and Australia[40,41,42]. Furthermore, there is no correlation of the sample weight with Pb isotope composition and/or with the magnitude of Pb isotope offset between the two size fractions. Instead, the Pb isotope offset increases with decreasing difference in sample weight as well as with decreasing difference in Pb concentrations between the two surface sample size fractions (see Supplementary Data File 3). The Pb concentrations in our samples show good correlation with other trace metal concentrations, and in particular with Zr content indicative of lithogenic input (LGM open ocean samples: $R^2 = 0.74$, n = 73; Holocene open ocean samples: $R^2 = 0.92$, n = 11; see Supplementary Data Files 1 and 3). These systematic characteristics appear inconsistent with Pb isotope shifts randomly induced by sample heterogeneity and/or lab contamination. In addition, it would remain elusive why the Pb isotope offset is limited to the coretop samples, but largely absent in the LGM samples (Supplementary Fig. 8). Therefore, we invoke other explanations for the observed offset between <5 μm and <10 μm fraction sediments in the mid-latitude South Pacific.

Previous work showed that industrial activities, such as coal burning, mining and metal production released substantial amounts of Pb into the atmosphere[41,43,44,45]. A prominent example is the Broken Hill ore deposit in Australia which supplied remarkably unradiogenic Pb as an additive to leaded gasoline until the 1990s[41,45,46]. The Pb adsorbed to particulates would be removed during our leaching procedure. Yet, industrial activities and combustion also release (mineral) particles, in particular during (open cut) mining, metal processing and/or coal combustion[43,44,45,47,48]. Peat bog records from the Snowy Mountains in Southeast Australia show a shift to unradiogenic Pb isotope compositions in the mineral fraction, which has been ascribed to the beginning/intensification of mining, smelting and coal combustion in Australia around 1880[41]. Around the same time, Antarctic dust fallout at Coats Land (Weddell Sea, East Antarctica) showed increased contributions of unradiogenic Pb[49]. The Pb isotope shift at Coats Land was attributed to particles released by anthropogenic activities such as Pb mining, smelting and/or coal combustion in the Southern Hemisphere, and in particular in Australia[49]. In addition, particles released in the Northern Hemisphere (natural and anthropogenic) can reach the Southern Hemisphere and potentially deliver unradiogenic particulate Pb to the mid-latitude South Pacific[46,50]. The absence of a pronounced Pb isotope offset between the <5 μm and <10 μm

fractions of LGM sediments would also suggest that the effect is related to anthropogenic activities.

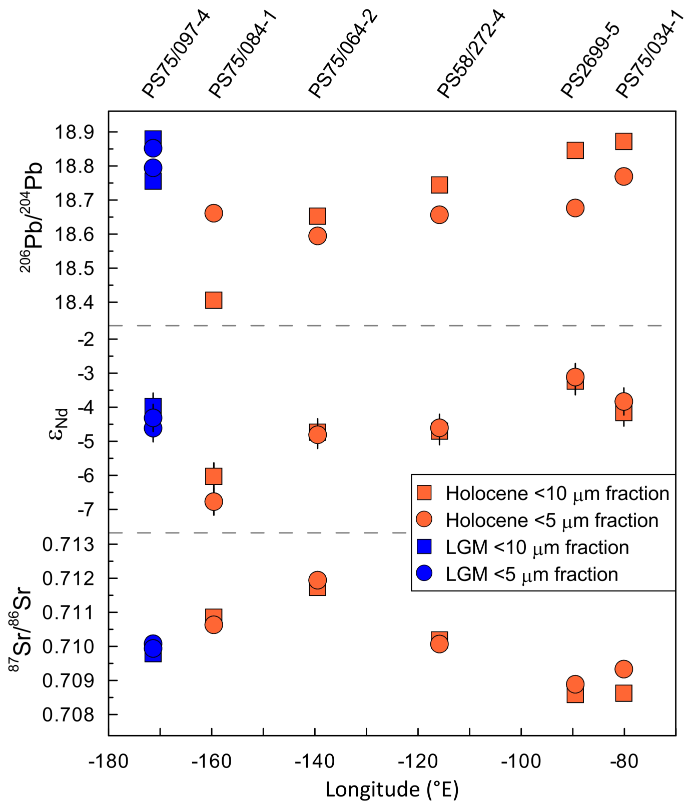

Supplementary Figure 8: Radiogenic isotope results for sedimentary <5 µm and <10 µm grain size fractions from the South Pacific. All results were obtained from the same sample material and subjected to the same physical and chemical preparation (see Methods section of the main text for details). Note the modest Sr isotope difference and the pronounced Pb isotope disparity in the respective Holocene sediment fractions. The error bars represent the 2SD of repeated analyses of secondary rock reference materials as reported in the Methods section of the main text. No error bars are shown where the 2SD is smaller than the symbol size.

Therefore, we entertain the idea that a higher abundance of far-travelled (anthropogenic) fine mineral particles induced the systematically lower $^{206}Pb/^{204}Pb$ (other Pb isotope ratios accordingly) in the <5 µm fraction of our South Pacific surface sediments. Anthropogenic emissions changed in character and magnitude during the late Holocene[17,41,46,49] so that the different ages of the surface sediments[2] may explain some of the site-specific Pb isotope offsets between the respective size fractions. Another controlling factor could be the difference in atmospheric lifetime of the two size fractions[51] and hence, the distance of the South Pacific core locations to the source(s) of emissions. Importantly, a high Pb isotope offset between the two fractions would be indicative of a relatively increased abundance of anthropogenic particles in a given sample. If such particles would also have a pronounced effect on the Pb isotope budget of the <10 µm fraction, we would expect decreasing $^{206}Pb/^{204}Pb$ in the <10 µm fraction with increasing Pb isotope offsets to the <5 µm fraction. As this is not the case, our observations do not

contradict previous conclusions[2] that the <10 µm lithogenic fraction of South Pacific surface sediments reflects predominantly natural Southern Hemisphere dust input.

Supplementary Figure 9: Austral wintertime particle trajectories from Central South America

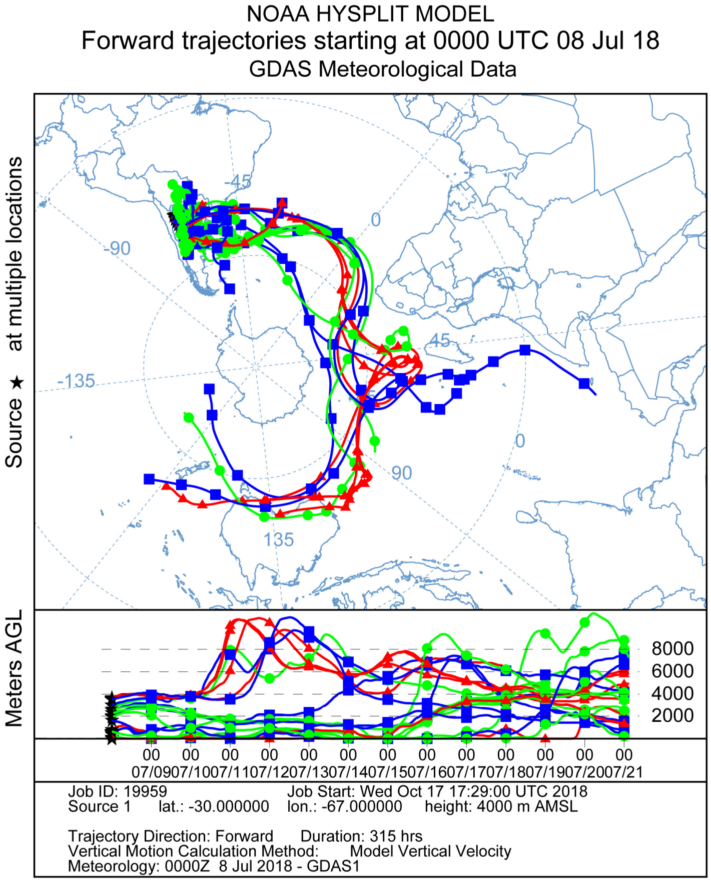

Supplementary Figure 9: Hybrid Single Particle Lagrangian Integrated Trajectory (HYSPLIT) forward air parcel simulation for austral wintertime (July 2018). The particles were released at multiple locations (n = 24) over Central South America in a grid between 25.0°S/66.1°W and 30.0°S/67.0°W in 4000 m above mean sea-level (AMSL). The trajectories were simulated for 315 hours starting from 8th July 2018 using Global Data Assimilation System (GDAS) dataset. AGL: above ground level. Note that the subtropical jet stream is centered at ~7 km over South America[73].

Supplementary References

1. Jochum, K. P. *et al.* Reference values following ISO guidelines for frequently requested rock reference materials. *Geostand. Geoanal. Res.* **40,** 333–350 (2016).
2. Wengler, M. *et al.* A geochemical approach to reconstruct modern dust fluxes and sources to the South Pacific. *Geochim. Cosmochim. Acta* **264**, 205–223 (2019).
3. Weis, D. *et al.* High-precision isotopic characterization of USGS reference materials by TIMS and MC-ICP-MS. *Geochem. Geophys. Geosyst.* **7,** Q08006 (2006).
4. Raczek, I., Stoll, B., Hofmann, A. W. & Jochum, K. P. High-precision trace element data for the USGS reference materials BCR-1, BCR-2, BHVO-1, BHVO-2, AGV-1, AGV-2, DTS-1, DTS-2, GSP-1 and GSP-2 by ID-TIMS and MIC-SSMS. *Geostand. Newsl.* **25,** 77–86 (2001).
5. Gutjahr, M. *et al.* Reliable extraction of a deepwater trace metal isotope signal from Fe–Mn oxyhydroxide coatings of marine sediments. *Chem. Geol.* **242,** 351–370 (2007).
6. Carroll, D. & Starkey, H. C. Reactivity of clay minerals with acids and alkalies. *Clays Clay Miner.* **19,** 321–333 (1971).
7. Berner, R. A., Sjöberg, E. L., Velbel, M. A. & Krom, M. D. Dissolution of pyroxenes and amphiboles during weathering. *Science* **207,** 1205–1206 (1980).
8. Berner, R. A. & Holdren, G. R. Mechanism of feldspar weathering—II. Observations of feldspars from soils. *Geochim. Cosmochim. Acta* **43,** 1173–1186 (1979).
9. Toyoda, K., Nakamura, Y. & Masuda, A. Rare earth elements of Pacific pelagic sediments. *Geochim. Cosmochim. Acta* **54,** 1093–1103 (1990).
10. Bradtmiller, L. I., Anderson, R. F., Fleisher, M. Q. & Burckle, L. H. Comparing glacial and Holocene opal fluxes in the Pacific sector of the Southern Ocean. *Paleoceanography* **24,** (2009).
11. Benz, V., Esper, O., Gersonde, R., Lamy, F. & Tiedemann, R. Last Glacial Maximum sea surface temperature and sea-ice extent in the Pacific sector of the Southern Ocean. *Quat. Sci. Rev* **146,** 216–237 (2016).
12. Molina-Kescher, M., Frank, M. & Hathorne, E. C. Nd and Sr isotope compositions of different phases of surface sediments in the South Pacific: extraction of seawater signatures, boundary exchange, and detrital/dust provenance. *Geochem. Geophys. Geosyst.* **15,** 3502–3520 (2014).
13. Molina-Kescher, M. *et al.* Reduced admixture of North Atlantic Deep Water to the deep central South Pacific during the last two glacial periods. *Paleoceanography* **31,** 651–668 (2016).
14. Jweda, J., Bolge, L., Class, C. & Goldstein, S. L. High precision Sr-Nd-Hf-Pb isotopic compositions of USGS reference material BCR-2. *Geostand. Geoanal. Res.* **40,** 101–115 (2016).
15. Gili, S. *et al.* Glacial/interglacial changes of Southern Hemisphere wind circulation from the geochemistry of South American dust. *Earth Planet. Sci. Lett.* **469,** 98–109 (2017).
16. Taylor, S. & McLennan, S. *The Continental Crust: Its Composition and Evolution* (Blackwell, Oxford, 1985).
17. Vallelonga, P. *et al.* Lead isotopic compositions in the EPICA Dome C ice core and Southern Hemisphere potential source areas. *Quat. Sci. Rev.* **29,** 247–255 (2010).
18. Garçon, M., Chauvel, C., France-Lanord, C., Limonta, M. & Garzanti, E. Which minerals control the Nd–Hf–Sr–Pb isotopic compositions of river sediments? *Chem. Geol.* **364,** 42–55 (2014).
19. Carter, L. & Mitchell, J. S. Late Quaternary sediment pathways through the deep ocean, east of New Zealand. *Paleoceanography* **2,** 409–422 (1987).
20. Mccave, I. N. & Carter, L. Recent sedimentation beneath the Deep Western Boundary Current off northern New Zealand. *Deep Sea Res. Pt. 1 Oceanogr. Res. Pap.* **44,** 1203–1237 (1997).
21. Li, F., Ginoux, P. & Ramaswamy, V. Transport of Patagonian dust to Antarctica. *J. Geophys. Res. Atmos.* **115,** (2010).
22. McNutt, R. H. Strontium isotopes. In *Environmental Tracers in Subsurface Hydrology* (eds. Cook, P. G. & Herczeg, A. L.), 233–260 (Springer, Boston, 2000).
23. Clauer, N. Relationship between the isotopic composition of strontium in newly formed continental clay minerals and their source material. *Chem. Geol.* **27,** 115–124 (1979).

24. Blum, J. D. & Erel, Y. Radiogenic isotopes in weathering and hydrology. In *Treatise on Geochemistry* (eds. Holland H. D. & Turekian, K. K.), 365–392 (Pergamon, Oxford, 2003).

25. Grousset, F. E. *et al.* Antarctic (Dome C) ice-core dust at 18 k.y. B.P.: isotopic constraints on origins. *Earth Planet. Sci. Lett.* **111,** 175–182 (1992).

26. Delmonte, B. *et al.* Dust size evidence for opposite regional atmospheric circulation changes over east Antarctica during the last climatic transition. *Clim. Dyn.* **23,** 427–438 (2004).

27. Grousset, F. E. & Biscaye, P. E. Tracing dust sources and transport patterns using Sr, Nd and Pb isotopes. *Chem. Geol.* **222,** 149–167 (2005).

28. Feng, J.-L., Zhu, L.-P., Zhen, X.-L. & Hu, Z.-G. Grain size effect on Sr and Nd isotopic compositions in eolian dust: implications for tracing dust provenance and Nd model age. *Geochem. J.* **43,** 123–131 (2009).

29. Meyer, I., Davies, G. R. & Stuut, J.-B. W. Grain size control on Sr-Nd isotope provenance studies and impact on paleoclimate reconstructions: an example from deep-sea sediments offshore NW Africa. *Geochem. Geophys. Geosyst.* **12,** (2011).

30. Gili, S. *et al.* Provenance of dust to Antarctica: a lead isotopic perspective. *Geophys. Res. Lett.* **43,** 2291–2298 (2016).

31. Khondoker, R. *et al.* New constraints on elemental and Pb and Nd isotope compositions of South American and Southern African aerosol sources to the South Atlantic Ocean. *Geochemistry* **78,** 372–384 (2018).

32. Hemming, S. R. *et al.* Strontium isotope tracing of terrigenous sediment dispersal in the Antarctic Circumpolar Current: Implications for constraining frontal positions. *Geochem. Geophys. Geosyst.* **8,** Q06N13 (2007).

33. Rackow, T. *et al.* A simulation of small to giant Antarctic iceberg evolution: differential impact on climatology estimates. *J. Geophys. Res. Oceans* **122,** 3170–3190 (2017).

34. Winton, V. H. L. *et al.* The origin of lithogenic sediment in the south-western Ross Sea and implications for iron fertilization. *Antarct. Sci.* **28,** 250–260 (2016).

35. Holland, P. R. & Kwok, R. Wind-driven trends in Antarctic sea-ice drift. *Nat. Geosci.* **5,** 872–875 (2012).

36. Lang Farmer, G., Licht, K., Swope, R. J. & Andrews, J. Isotopic constraints on the provenance of fine-grained sediment in LGM tills from the Ross Embayment, Antarctica. *Earth Planet. Sci. Lett.* **249,** 90–107 (2006).

37. Blakowski, M. A. *et al.* A Sr-Nd-Hf isotope characterization of dust source areas in Victoria Land and the McMurdo Sound sector of Antarctica. *Quat. Sci. Rev.* **141,** 26–37 (2016).

38. Gaiero, D. M., Probst, J.-L., Depetris, P. J., Bidart, S. M. & Leleyter, L. Iron and other transition metals in Patagonian riverborne and windborne materials: geochemical control and transport to the southern South Atlantic Ocean. *Geochim. Cosmochim. Acta* **67,** 3603–3623 (2003).

39. Gaiero, D. M., Depetris, P. J., Probst, J.-L., Bidart, S. M. & Leleyter, L. The signature of river- and wind-borne materials exported from Patagonia to the southern latitudes: a view from REEs and implications for paleoclimatic interpretations. *Earth Planet. Sci. Lett.* **219,** 357–376 (2004).

40. De Deckker, P. & Norman, M. D. Re-evaluation of the composition of sediments from the Murray Darling Basin of Australia as a potential source area for airborne dust to EPICA Dome C in Antarctica. Reply to Comment on “Lead isotopic evidence for an Australian source of aeolian dust to Antarctica at times over the last 170,000years” by P. De Deckker, M. Norman, I.D. Goodwin, A. Wain and F.X. Gingele [Palaeogeogr. Palaeoclimatol. Palaeoecol. 285 (2010) 205–223]. *Palaeogeogr. Palaeoclimatol. Palaeoecol.* **298,** 437–442 (2010).

41. Marx, S. K. & Kamber, B. S. Trace-element systematics of sediments in the Murray–Darling Basin, Australia: sediment provenance and palaeoclimate implications of fine scale chemical heterogeneity. *Appl. Geochem.* **25,** 1221–1237 (2010).

42. Marx, S. K., Kamber, B. S., McGowan, H. A. & Zawadzki, A. Atmospheric pollutants in alpine peat bogs record a detailed chronology of industrial and agricultural development on the Australian continent. *Environ. Pollut.* **158,** 1615–1628 (2010).

43. Nriagu, J. O. & Pacyna, J. M. Quantitative assessment of worldwide contamination of air, water and soils by trace metals. *Nature* **333,** 134–139 (1988).

44. Mukai, H. *et al.* Characterization of sources of lead in the urban air of Asia using ratios of stable lead isotopes. *Environ. Sci. Technol.* **27,** 1347–1356 (1993).
45. Chiaradia, M., Chenhall, B. E., Depers, A. M., Gulson, B. L. & Jones, B. G. Identification of historical lead sources in roof dusts and recent lake sediments from an industrialized area: indications from lead isotopes. *Sci. Total Environ.* **205,** 107–128 (1997).
46. Bollhöfer, A. & Rosman, K. J. R. Isotopic source signatures for atmospheric lead: the Southern Hemisphere. *Geochim. Cosmochim. Acta* **64,** 3251–3262 (2000).
47. Davison, R. L., Natusch, D. F. S., Wallace, J. R. & Jr, C. A. E. Trace elements in fly ash. Dependence of concentration on particle size. *Environ. Sci. Technol.* **8,** 1107– 1113 (1974).
48. Saarnio, K. *et al.* Chemical composition and size of particles in emissions of a coal-fired power plant with flue gas desulfurization. *J. Aerosol Sci.* **73,** 14–26 (2014).
49. Planchon, F. A. M. *et al.* One hundred fifty–year record of lead isotopes in Antarctic snow from Coats Land. *Geochim. Cosmochim. Acta* **67,** 693–708 (2003).
50. Li, F., Ginoux, P. & Ramaswamy, V. Distribution, transport, and deposition of mineral dust in the Southern Ocean and Antarctica: contribution of major sources. *J. Geophys. Res. Atmos.* **113,** D10207 (2008).
51. Kok, J. F. *et al.* Smaller desert dust cooling effect estimated from analysis of dust size and abundance. *Nat. Geosci.* **10,** 274–278 (2017).
52. Revel-Rolland, M. *et al.* Eastern Australia: A possible source of dust in East Antarctica interglacial ice. *Earth Planet. Sci. Lett.* **249,** 1–13 (2006).
53. McGowan, H. A., Kamber, B., McTainsh, G. H. & Marx, S. K. High resolution provenancing of long travelled dust deposited on the Southern Alps, New Zealand. *Geomorphology* **69,** 208–221 (2005).
54. De Deckker, P. Airborne dust traffic from Australia in modern and Late Quaternary times. *Glob. Planet. Change* **184,** 103056 (2020).
55. Gingele, F. X. & De Deckker, P. Clay mineral, geochemical and Sr–Nd isotopic fingerprinting of sediments in the Murray–Darling fluvial system, southeast Australia. *Aust. J. Earth Sci.* **52,** 965–974 (2005).
56. De Deckker, P., Norman, M., Goodwin, I. D., Wain, A. & Gingele, F. X. Lead isotopic evidence for an Australian source of aeolian dust to Antarctica at times over the last 170,000 years. *Palaeogeogr. Palaeoclimatol. Palaeoecol.* **285,** 205–223 (2010).
57. Gingele, F., De Deckker, P. & Norman, M. Late Pleistocene and Holocene climate of SE Australia reconstructed from dust and river loads deposited offshore the River Murray Mouth. *Earth Planet. Sci. Lett.* **255,** 257–272 (2007).
58. Marx, S. K., Kamber, B. S. & McGowan, H. A. Provenance of long-travelled dust determined with ultra-trace-element composition: a pilot study with samples from New Zealand glaciers. *Earth Surf. Process. Landf.* **30,** 699–716 (2005).
59. Noble, T. L. *et al.* Greater supply of Patagonian-sourced detritus and transport by the ACC to the Atlantic sector of the Southern Ocean during the last glacial period. *Earth Planet. Sci. Lett.* **317–318,** 374–385 (2012).
60. Sugden, D. E., McCulloch, R. D., Bory, A. J.-M. & Hein, A. S. Influence of Patagonian glaciers on Antarctic dust deposition during the last glacial period. *Nat. Geosci.* **2,** 281–285 (2009).
61. Simões Pereira, P. *et al.* Geochemical fingerprints of glacially eroded bedrock from West Antarctica: detrital thermochronology, radiogenic isotope systematics and trace element geochemistry in Late Holocene glacial-marine sediments. *Earth-Sci. Rev.* **182,** 204–232 (2018).
62. Delmonte, B. *et al.* Causes of dust size variability in central East Antarctica (Dome B): atmospheric transport from expanded South American sources during Marine Isotope Stage 2. *Quat. Sci. Rev.* **168,** 55–68 (2017).
63. Pichat, S., Abouchami, W. & Galer, S. J. G. Lead isotopes in the Eastern Equatorial Pacific record Quaternary migration of the South Westerlies. *Earth Planet. Sci. Lett.* **388,** 293–305 (2014).
64. GLIMS and NSIDC (2019): Global land ice measurements from space glacier database. Compiled and made available by the international GLIMS community and the National Snow and Ice Data Center, Boulder CO, U.S.A. doi:10.7265/N5V98602
65. Schlitzer, R. Ocean Data View, odv.awi.de (2019).

66. Klinkhammer, G. P., Elderfield, H., Edmond, J. M. & Mitra, A. Geochemical implications of rare earth element patterns in hydrothermal fluids from mid-ocean ridges. *Geochim. Cosmochim. Acta* **58,** 5105–5113 (1994).

67. Dulski, P. Reference materials for geochemical studies: new analytical data by ICP-MS and critical discussion of reference values. *Geostand. Newsl.* **25,** 87–125 (2001).

68. Guichard, F., Church, T. M., Treuil, M. & Jaffrezic, H. Rare earths in barites: distribution and effects on aqueous partitioning. *Geochim. Cosmochim. Acta* **43,** 983–997 (1979).

69. Haley, B. A., Klinkhammer, G. P. & Mix, A. C. Revisiting the rare earth elements in foraminiferal tests. *Earth Planet. Sci. Lett.* **239,** 79–97 (2005).

70. Douglas, G. B., Gray, C. M., Hart, B. T. & Beckett, R. A strontium isotopic investigation of the origin of suspended particulate matter (SPM) in the Murray-Darling River system, Australia. *Geochim. Cosmochim. Acta* **59,** 3799–3815 (1995).

71. Hart, S. R. A large-scale isotope anomaly in the Southern Hemisphere mantle. *Nature* **309,** 753–757 (1984).

72. Borunda, A. *Tracing dust in the Southern Hemisphere over the last glacial cycle* (Columbia University, 2019).

73. Gaiero, D. M. *et al.* Ground/satellite observations and atmospheric modeling of dust storms originating in the high Puna-Altiplano deserts (South America): implications for the interpretation of paleo-climatic archives. *J. Geophys. Res. Atmos.* **118,** 3817–3831 (2013).
