# Supplementary material for: A circumpolar dust conveyor in the glacial Southern Ocean
Source: Nat Commun. 2020 Nov 9;11:5655. doi: 10.1038/s41467-020-18858-y (PMC7652835; doi:10.1038/s41467-020-18858-y)
Supplement: Supplementary file 5 — Description of additional supplementary files [file 41467_2020_18858_MOESM5_ESM.pdf]

# Description of Supplementary Data Files

**File name: Supplementary Data File 1**

**Description: Results of Last Glacial Maximum dust fraction samples (<5 μm) from the South Pacific.**

**File name: Supplementary Data File 2**

**Last Glacial Maximum average values calculated from individual sample results reported in Supplementary Data File 1.**

**File name: Supplementary Data File 3**

**Description: Results of Holocene dust fraction samples (<5/10 μm) from the South Pacific.**
